# Dose-response Relationships Between Cigarette Smoking and Breast Cancer Risk: A Systematic Review and Meta-analysis

**DOI:** 10.2188/jea.JE20220206

**Published:** 2023-12-05

**Authors:** Marco Scala, Cristina Bosetti, Vincenzo Bagnardi, Irene Possenti, Claudia Specchia, Silvano Gallus, Alessandra Lugo

**Affiliations:** 1 Department of Medical Epidemiology, Istituto di Ricerche Farmacologiche Mario Negri IRCCS, Milan, Italy; 2 Department of Statistics and Quantitative Methods, Università degli Studi di Milano-Bicocca, Milan, Italy; 3 Department of Molecular and Translational Medicine, Università degli Studi di Brescia, Brescia, Italy

**Keywords:** breast cancer, meta-analysis, risk, systematic review, tobacco smoking

## Abstract

**Background:**

The possible association between cigarette smoking and breast cancer risk has been quite controversial.

**Methods:**

We conducted a systematic review and meta-analysis of all available observational studies published on the issue up to January 2020. Random-effects models were used to compute pooled relative risks (RRs) for cigarette smoking status and dose-risk relationships were evaluated using one-stage random-effects dose-response models.

**Results:**

A total of 169 studies were selected, providing a pooled RR for breast cancer of 1.07 (95% confidence interval [CI], 1.05–1.10) for current, 1.08 (95% CI, 1.06–1.10) for former, and 1.09 (95% CI, 1.07–1.11) for ever smokers, compared to never smokers. Results were consistent in case-control and cohort studies. No meaningful differences were observed across strata of most covariates considered, nor according to relevant genetic mutations and polymorphisms (ie, BRCA mutation, N-acetyltransferase and glutathione S-transferase genotypes, and P53). Breast cancer risk increased linearly with intensity of smoking (RR 1.12; 95% CI, 1.08–1.16 for 20 cigarettes/day and 1.26; 95% CI, 1.17–1.36 for 40 cigarettes/day), and with increasing duration of smoking (RR 1.05; 95% CI, 1.03–1.08 for 20 years of smoking and 1.11; 95% CI, 1.06–1.16 for 40 years of smoking).

**Conclusion:**

The present large and comprehensive meta-analysis—conducted using an innovative approach for study search—supports the evidence of a causal role of tobacco smoking on breast cancer risk.

## INTRODUCTION

Breast cancer is the most commonly diagnosed cancer and the leading cause of cancer death among women worldwide.^1^ In 2020, there were about 2.26 million newly diagnosed female cases—accounting for almost 1 in 4 cancer cases among women—and 685,000 female deaths from breast cancer (ie, 15% of total cancer deaths).^2^

The onset of female breast cancer can be caused by various environmental and genetic factors. Some of these risk factors are not modifiable, such as age, familiarity, and hormonal factors, while others are modifiable, such as smoking and alcohol consumption.^3^

The possible association between cigarette smoking and the risk of breast cancer has been quite controversial. In 1986, the International Agency for Research on Cancer (IARC) reported that smoking could reduce the risk for breast cancer possibly due to its antiestrogenic effect, although the evidence was inconsistent.^4^ A subsequent IARC publication reported that most epidemiological studies have found no association with active smoking, after controlling for established risk factors (including in particular alcohol drinking, which represents a strong confounding factor).^5^ However, in 2012 the IARC—on the basis of about 130 observational studies—concluded that there was a direct association between tobacco smoking and female breast cancer risk.^6^ Consistently, one of the most recent and comprehensive meta-analysis concluded that the evidence accumulated over the last years indicates that active tobacco smoking is associated with a modest, but real increase in the risk of breast cancer.^7^

With reference to possible differences in risk according to menopausal status and oestrogen receptor (ER) status, in 2012 the IARC indicated that studies were quite limited and inconsistent.^6^ The meta-analysis by Macacu et al indicated that the risks were similar in pre and post-menopausal women.^7^ A few papers suggested a possible stronger effect of smoking on breast cancer in BRCA mutation carriers or women who have a genetic reduced inactivation of tobacco carcinogens—such as the N-acetyltransferase (NAT) and glutathione S-transferase (GST) M1—but the evidence was limited and not conclusive.^8^^–^^12^

In this meta-analysis, using an innovative approach for the identification of original articles,^13^ we aim to verify and quantify the association between cigarette smoking and female breast cancer risk, with a focus on dose-response relationships and possible differential role of tobacco smoking according to relevant genetic mutations and polymorphisms.

## METHODS

The present work is part of a series of systematic reviews and meta-analyses on the association between cigarette smoking and the risk of cancer.^13^^–^^18^ It takes advantage of an innovative methodology to identify original articles, based on a combination of umbrella and traditional reviews, that has already been described in previous publications.^13^^,^^14^ The study protocol has been registered in the International Prospective Register of Systematic Reviews (PROSPERO; registration number: CRD42017063991). As this was not an individual patient-level meta-analysis, institutional review board permission and informed consent were not required.

### Search strategy

In the first step, we conducted an umbrella review on smoking and the risk of cancer through a comprehensive literature search on various databases (PubMed/MEDLINE, Embase, Institute for Scientific Information Web of Science, and the Cochrane Database of Systematic Reviews) to identify all meta-analyses, pooled analyses, and reviews on the association between cigarette smoking and the risk of cancer at any site. The search was run on April 27, 2017 and then updated on January 14, 2020.^13^ We identified eight systematic reviews/meta-analyses^7^^,^^19^^–^^25^ and five pooled analyses^26^^–^^30^ on tobacco smoking and breast cancer (eFigure 1). We also considered two monographs of the IARC on tobacco smoking,^5^^,^^6^ three reports from the Centers for Disease Control and Prevention,^31^^–^^33^ and two reports from the Canadian Expert Panel on Tobacco smoke and Breast Cancer Risk.^34^^,^^35^ We screened the 20 above-mentioned reports to identify original publications on tobacco smoking and the risk of breast cancer.

In the second step, we carried out a literature search on PubMed/MEDLINE and Embase to identify all original studies published between 2015 (ie, the publication date of the last and most comprehensive review available on the topic^7^) and June 30, 2020. The search string included combinations of MeSH terms and text words related to breast cancer and tobacco or smoking (eMaterial 1). After the exclusion of duplicate publications and ineligible articles and the inclusion of 16 additional relevant publications identified from other sources, the update of the scientific literature resulted in 76 additional original publications on cigarette smoking and the risk of breast cancer. Combining original articles identified in the umbrella review (step 1) and in the update of the literature (step 2), we retrieved 371 non-duplicate original publications (eFigure 1) that were screened for eligibility on the basis of their full text using the eligibility criteria described below. Data search and screening were made by two independent reviewers.

### Eligibility criteria

Studies were included in the present meta-analysis if they satisfied the following eligibility criteria: i) they were either case-control studies (including nested case-control studies or pooled analyses of case-control studies) or cohort studies (including case-cohort studies or pooled analyses of cohort studies); ii) they were published as original articles in English; iii) they provided data on the general female population; iv) they provided information on the association between cigarette smoking and the risk of female malignant breast cancer; v) they reported risk estimates, including risk ratios, odds ratios, hazard ratios, or mortality rate ratios—all referred to as relative risks (RRs)—for at least one variable among smoking status (current, former and/or ever smoking), intensity, duration and time since quitting, compared to never or current cigarette smokers, and corresponding 95% confidence intervals (CIs), or providing sufficient information to compute them.

### Data extraction

For each eligible study, we collected general information on the publication (eg, first author, year of publication, and journal), study (eg, country, study name, calendar period, study design, and sample size), the model used for RR estimates (including covariates allowed for), and RRs with the corresponding 95% CIs and, when available, the number of cases and controls (or subjects at risk/person years for cohort studies) for various exposure categories.

Where necessary, we used the Hamling method^36^ in order to change the reference category or to collapse the RRs of two or more categories with the same reference group. When various RRs were reported separately for different types of breast cancer, we used the method described by Rucker and colleagues^37^ to obtain a single RR for overall breast cancer.

### Statistical analysis

Pooled RRs for current, former, and ever smokers vs never smokers were estimated for breast cancer, overall and by study design (ie, cohort and case-control). These estimates were obtained using random-effects models to take into account the heterogeneity of risk estimates.^38^ Heterogeneity between studies was assessed using the χ^2^ test, and inconsistency was measured using the I^2^ statistic, which represents the proportion of total variation attributable to between-study variance.^39^ We conducted stratified analyses based on various study, population, and cancer characteristics (study design, geographic area, type of control, endpoint, year of publication, menopausal status, and ER status); analyses stratified by relevant genetic polymorphisms and mutations (ie, BRCA, NAT2, GSTM1, P53) were also conducted. To evaluate publication bias, we examined the funnel plots^40^ and applied the Egger’s test for funnel plot asymmetry.^41^

We evaluated dose-response relationships between smoking variables (ie, smoking intensity, smoking duration, and time since quitting) and log RR of breast cancer, either linear or not, using one-stage random-effects dose-response models^42^ (eMaterial 2). For each exposure variable, we tested the statistical significance of nonlinear coefficients using the Wald test. In case of rejection of linearity, non-linear relationships were modelled using restricted cubic spline with three knots at fixed percentiles of exposure (10%, 50%, and 90%).^14^^,^^43^ For each category, the level of exposure was assigned as the midpoint between the upper and the lower bounds; for open-ended upper categories, the level of exposure was determined as 1.2 times the lower bound.^13^^,^^44^^,^^45^ When the number of cases and/or controls in one or more exposure categories was not provided in the original study publication, we estimated the covariance among the log RR by considering the total number of cases and/or controls weighted by the average percent distribution of subjects pooled from all other studies.^46^

All statistical analyses were performed using the R-software version 3.4.1 (R Foundation for Statistical Computing, Vienna, Austria), and, in particular, the “meta” and “dosresmeta” packages.^46^

## RESULTS

### Study selection and description

Among 371 original publications, 211 original articles met the eligibility criteria (eFigure 1). After excluding 43 articles that provided duplicated data (eTable 1), 168 articles (for a total of 169 studies, since one article includes data for a case-control study and a cohort study^10^) were included in the present meta-analysis. The main characteristics of the case-control (*n* = 115) and cohort (*n* = 54) studies are summarized respectively in eTable 2 and eTable 3. The studies were published between 1986 and 2020 and included more than 400,000 breast cancer cases. Ninety studies provided a measure of the association for current smokers, 86 for former smokers, and 136 for ever smokers as compared to never smokers; 77 studies reported RR estimates for smoking intensity (31 among current smokers), 55 for smoking duration (11 among current smokers), and 17 for time since quitting smoking. Publications containing data that were partially excluded from the present meta-analysis, with the corresponding reasons of exclusion, are described in eTable 4.

### Quantitative data synthesis

**Figure 1.**
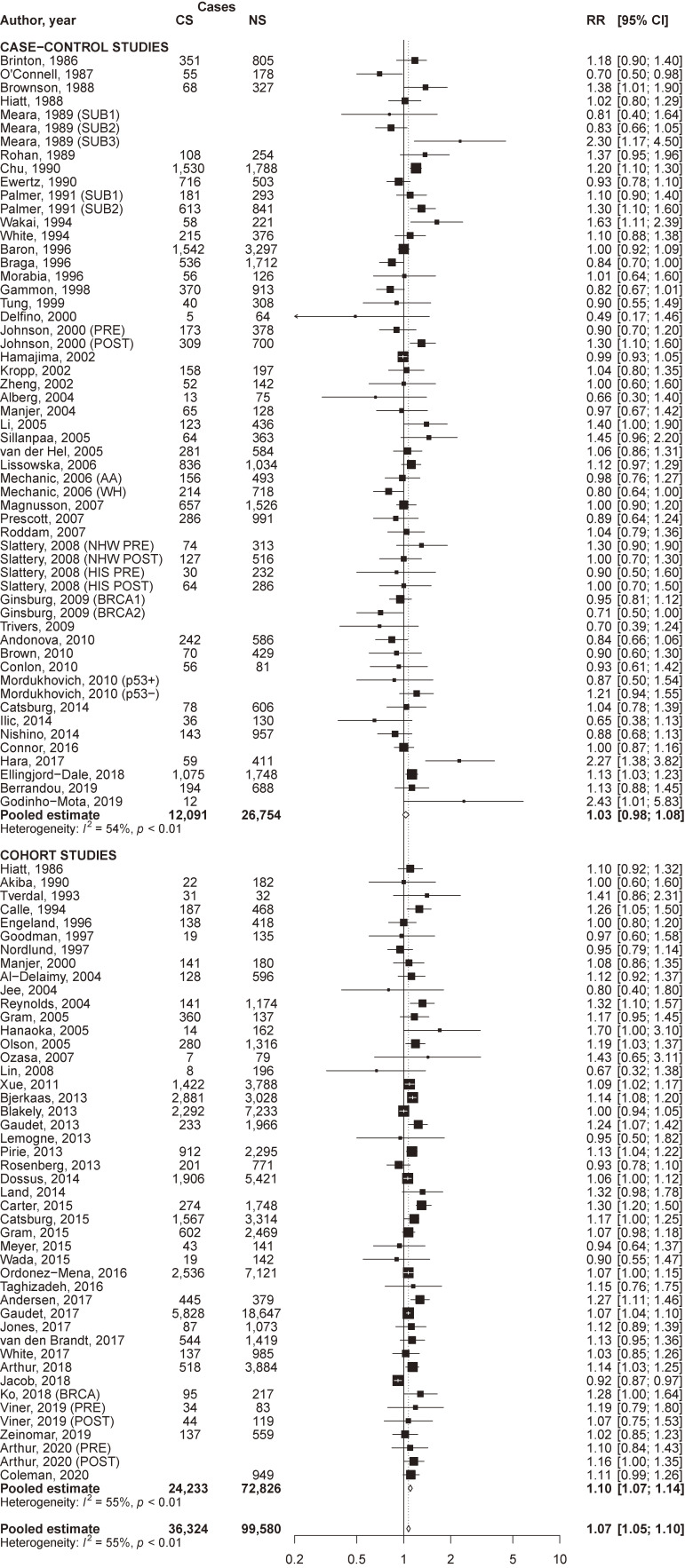
Forest plot of study-specific and pooled relative risk (RR) of breast cancer for current cigarette smokers (CS) versus never smokers (NS), by study design. AA, African-American ethnicity; BRCA, BRCA mutation; BRCA1, BRCA1 mutation; BRCA2, BRCA2 mutation; CI, confidence interval; HIS, Hispanic ethnicity; NHW, non-Hispanic Caucasian ethnicity; p53−, p53 negative; p53+, p53 positive; POST, post-menopausal women; PRE, pre-menopausal women; SUB1, first subgroup; SUB2, second subgroup; SUB3, third subgroup; WH, white ethnicity.

**Figure 2.**
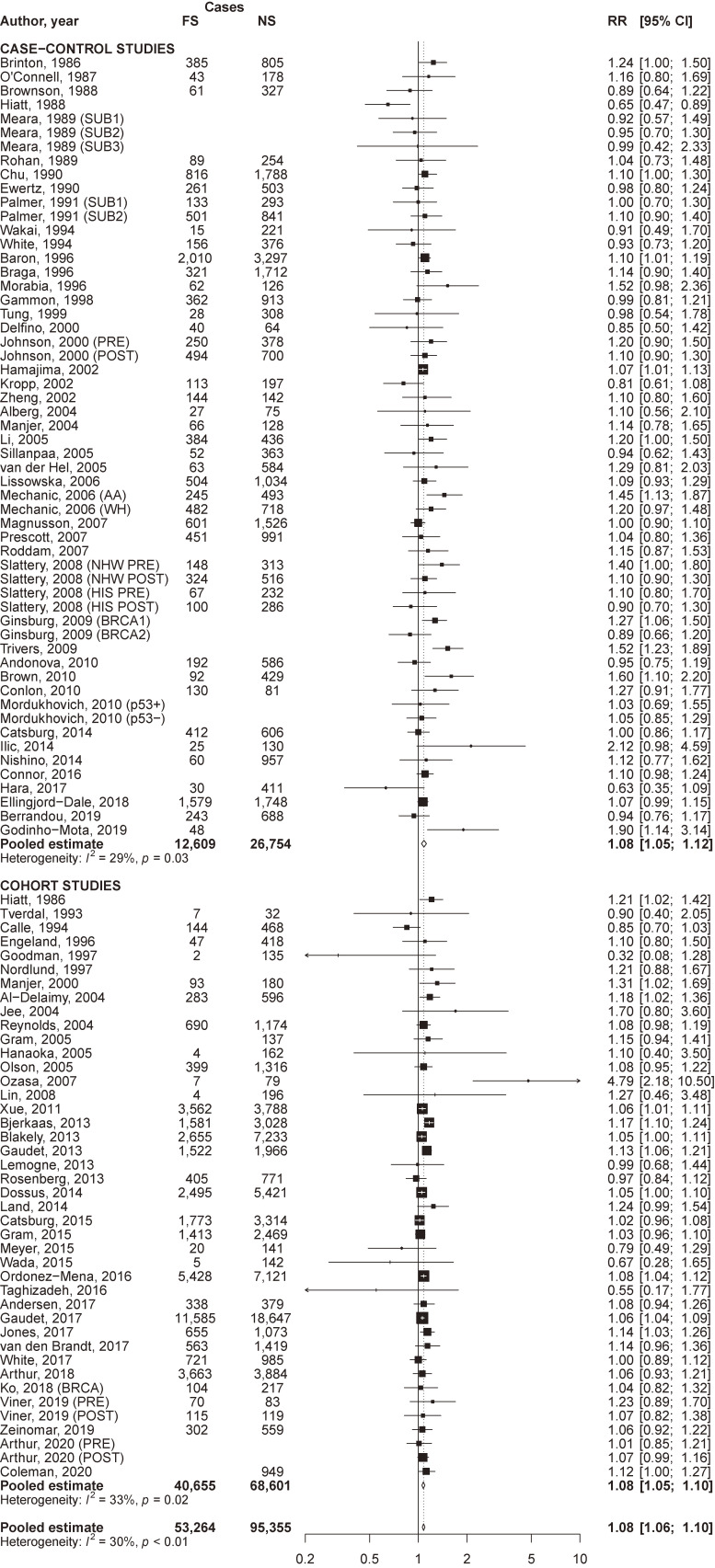
Forest plot of study-specific and pooled relative risk (RR) of breast cancer for former cigarette smokers (FS) versus never smokers (NS), by study design. AA, African-American ethnicity; BRCA, BRCA mutation; BRCA1, BRCA1 mutation; BRCA2, BRCA2 mutation; CI, confidence interval; HIS, Hispanic ethnicity; NHW, non-Hispanic Caucasian ethnicity; p53−, p53 negative; p53+, p53 positive; POST, post-menopausal women; PRE, pre-menopausal women; SUB1, first subgroup; SUB2, second subgroup; SUB3, third subgroup; WH, white ethnicity.

The pooled RR of breast cancer for current compared to never smokers was 1.07 (95% CI, 1.05–1.10) overall, and was significantly higher in cohort (RR 1.10; 95% CI, 1.07–1.14) than in case-control (RR 1.03; 95% CI, 0.98–1.08) studies (*P* of heterogeneity between study designs = 0.02; Figure 1). The pooled RR for former compared to never smokers was 1.08 (95% CI, 1.06–1.10) overall, 1.08 (95% CI, 1.05–1.12) in case-control, and 1.08 (95% CI, 1.05–1.10) in cohort studies (Figure 2). Corresponding estimates for ever smokers were 1.09 (95% CI, 1.07–1.11), 1.10 (95% CI, 1.06–1.14), and 1.08 (95% CI, 1.06–1.10), respectively (eFigure 2). For all estimates, a significant between-study heterogeneity was observed.

Possible sources of heterogeneity were investigated through stratified analyses (Table 1). Significant differences in the association between current smokers and breast cancer risk were observed according to the endpoint for cohort studies (RRs were 1.09 among studies on incidence and 1.18 among studies on mortality; *P* = 0.01), menopausal status (RRs were 1.03 for premenopausal women and 1.13 for postmenopausal women; *P* = 0.02). Among former smokers, significant differences were observed according to income group (RRs were 1.96 for middle and 1.08 for high-income countries; *P* < 0.01). Among ever smokers, significant differences were observed according to geographic area, the associations being higher in studies conducted in Asia (RR 1.32) and in one study conducted in South America (RR 2.02). No meaningful differences emerged for the effect of smoking on breast cancer risk according to other covariates considered, including ER status.

**Table 1.** Pooled relative risk and corresponding 95% confidence interval for breast cancer risk for current, former, and ever cigarette smokers versus never cigarette smokers, overall and in strata of selected characteristics

Strata	Current smokers				Former smokers				Ever smokers			
		
	Number of studies	Pooled RR (95% CI)	*P*-value^a^	*P*-value^b^	Number of studies	Pooled RR (95% CI)	*P*-value^a^	*P*-value^b^	Number of studies	Pooled RR (95% CI)	*P*-value^a^	*P*-value^b^
Total	90	1.07 (1.05–1.10)		<0.01	86	1.08 (1.06–1.10)		<0.01	135	1.09 (1.07–1.11)		<0.01

Geographic area^c^												
North America	39	1.10 (1.05–1.14)	0.28	<0.01	38	1.09 (1.05–1.12)	0.20	0.01	54	1.08 (1.06–1.11)	<0.01	<0.01
Europe	31	1.05 (1.01–1.10)		<0.01	29	1.08 (1.05–1.11)		0.31	39	1.07 (1.02–1.12)		<0.01
Asia	11	1.13 (0.90–1.40)		0.02	10	1.09 (0.76–1.57)		<0.01	30	1.32 (1.16–1.50)		<0.01
Oceania	2	1.11 (0.83–1.48)		0.09	2	1.05 (1.00–1.11)		0.96	3	1.03 (0.99–1.08)		0.60
South America	1	2.43 (1.01–5.84)		–	1	1.90 (1.14–3.15)		–	1	2.02 (1.30–3.13)		–

Income group^d^												
High income	83	1.08 (1.04–1.11)	0.87	<0.01	79	1.08 (1.06–1.10)	<0.01	<0.01	114	1.08 (1.06–1.11)	0.07	<0.01
Middle income	2	1.20 (0.33–4.35)		0.01	2	1.96 (1.29–3.00)		0.82	14	1.44 (1.06–1.95)		<0.01

Study design												
Cohort	44	1.10 (1.07–1.14)	0.02	<0.01	40	1.08 (1.05–1.10)	0.69	0.02	44	1.08 (1.06–1.10)	0.36	0.02
Case-control	46	1.03 (0.98–1.08)		<0.01	46	1.08 (1.05–1.12)		0.03	92	1.10 (1.06–1.14)		<0.01

Type of controls^e^												
Hospital	9	0.97 (0.80–1.17)	0.44	<0.01	9	1.08 (0.94–1.23)	0.84	0.18	25	1.09 (0.95–1.24)	0.85	<0.01
Population	34	1.04 (0.99–1.10)		<0.01	34	1.09 (1.04–1.14)		<0.01	62	1.10 (1.06–1.15)		<0.01

Endpoint^f^												
Incidence	34	1.09 (1.06–1.13)	0.01	<0.01	33	1.07 (1.06–1.09)	0.82	0.34	37	1.08 (1.06–1.10)	0.14	0.02
Mortality	11	1.18 (1.12–1.24)		0.46	8	1.10 (0.90–1.33)		<0.01	8	1.14 (1.06–1.22)		0.29

Year of publication												
<2002	26	1.06 (0.99–1.13)	0.28	<0.01	25	1.06 (1.01–1.12)	0.18	0.16	47	1.06 (1.01–1.11)	0.33	<0.01
2002–2011	29	1.04 (0.99–1.10)		0.03	29	1.12 (1.07–1.17)		<0.01	41	1.11 (1.07–1.17)		<0.01
≥2012	35	1.09 (1.06–1.13)		<0.01	32	1.07 (1.01–1.12)		0.11	47	1.09 (1.06–1.11)		<0.01

Menopausal status												
Pre-	22	1.03 (0.99–1.08)	0.02	0.42	22	1.09 (1.02–1.15)	0.57	0.20	37	1.10 (1.04–1.16)	0.61	<0.01
Post-	26	1.13 (1.06–1.21)		<0.01	25	1.06 (1.03–1.10)		0.55	40	1.12 (1.08–1.17)		<0.01

Estrogen Receptor												
Positive	12	1.09 (1.02–1.16)	0.39	0.19	12	1.09 (1.04–1.14)	0.81	0.18	16	1.11 (1.05–1.16)	0.57	0.02
Negative	12	1.04 (0.96–1.13)		0.40	12	1.08 (0.97–1.19)		0.10	16	1.08 (1.00–1.16)		0.08

CI, confidence interval; RR, relative risk.

^a^*P*-value for heterogeneity across strata.

^b^*P*-value for heterogeneity within strata.

^c^Studies conducted in multiple countries from different geographic areas were not included. No studies from Africa.

^d^Studies conducted in multiple countries with different income were not included. No studies from low income countries.

^e^Type of controls for case-control studies only. Pooled analyses considering both studies with hospital and with population controls were not included.

^f^Endpoint for cohort studies only. Studies providing RRs for both incidence and mortality were considered in both categories.

### Analysis by genetic factors

Twenty studies reported the association between ever smoking and the risk of breast cancer stratified by genetic features of female breast cancer, including BRCA mutation (*n* = 6 studies), GSTM1 (*n* = 3), NAT2 (*n* = 9), and P53 (*n* = 3). Risk estimates were consistent in women with BRCA mutation (RR 1.02), in those with GSTM1_null (RR 1.10), GSTM1_present (RR 1.09), and with (RR 1.14) or without (RR 1.06) P53 gene expression, although risk estimates in all these groups were not statistically significant. Breast cancer risk was significantly increased in women with NAT2 slow polymorphism (RR 1.19; 95% CI, 1.09–1.30), but not in those with NAT2 fast polymorphism (RR 1.00; 95% CI, 0.86–1.17), although the two estimates were not significantly heterogeneous (eFigure 3).

### Dose-response analysis

**Figure 3.**
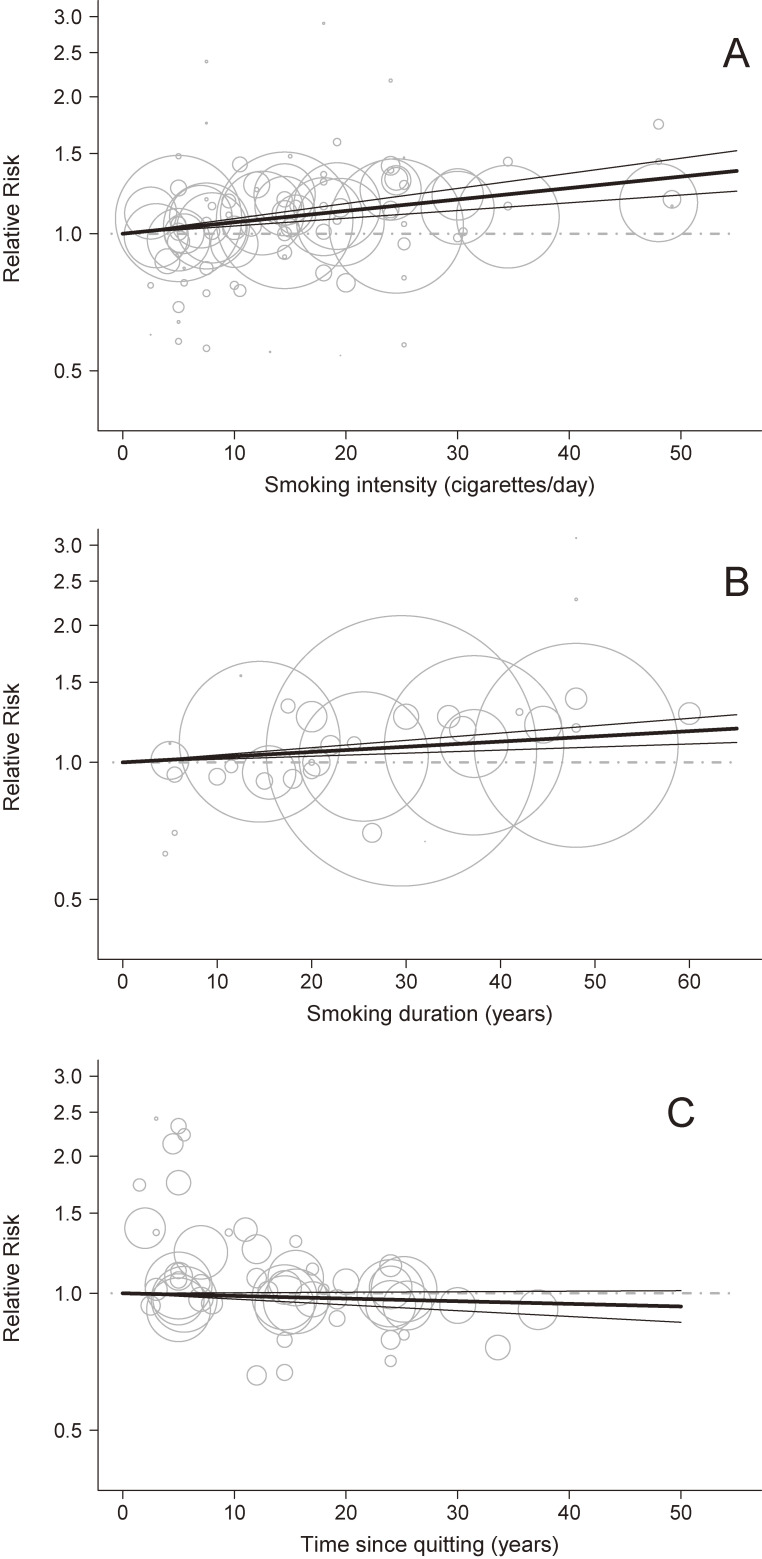
Relative risk (RR) for the dose-response relationships between cigarette smoking intensity, duration, and time since quitting and breast cancer. (**A**) cigarette smoking intensity (based on 31 studies); (**B**) cigarette smoking duration (based on 11 studies); (**C**) time since quitting (based on 17 studies). 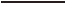
 Linear model from a random-effects dose-response model 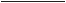
 95% confidence interval of the linear model 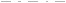
 RR for the reference category (never smokers in A and B, current smokers in C) 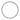
 RR for various exposure categories in each study included in the analysis. The area of the circle is proportional to the precision (ie, to the inverse variance) of the RR

Breast cancer risk increased linearly with intensity of smoking (RR 1.12; 95% CI, 1.08–1.16 for 20 cigarettes per day and 1.26; 95% CI, 1.17–1.36 for 40 cigarettes per day; Figure 3A). The RR of breast cancer also increased linearly with increasing duration of smoking: RRs were 1.05 (95% CI, 1.03–1.08) for 20 years of smoking and 1.11 (95% CI, 1.06–1.16) for 40 years of smoking (Figure 3B). There was no association between time since quitting and breast cancer risk, being 0.99 (95% CI, 0.97–1.00) after 10 years since quitting and 0.96 (95% CI, 0.92–1.01) after 30 years since smoking cessation (Figure 3C).

### Publication bias

No evidence of publication bias emerged for current and former smoking and breast cancer risk, either from visual inspection of the funnel plots (eFigure 4; panels A and B) or from the Egger’s test (*P* = 0.84 and *P* = 0.33 for current and former smokers, respectively); evidence for publication bias was, however, found for ever smokers (*P*-value for the Egger’s test = 0.01; eFigure 4; panel C).

## DISCUSSION

The present systematic review and meta-analysis gives the most up-to-date, exhaustive, and comprehensive assessment of the association between cigarette smoking and the risk of breast cancer. On the basis of 169 original studies, it provides further evidence of the causal role of tobacco smoking on breast cancer risk, with a 7%, 8%, and 9% increased risk in current, former, and ever smokers, respectively, and significant dose- and duration-risk relationships.

Our findings confirm the evidence from the last IARC Monograph^6^ and those reported by the last available comprehensive meta-analysis on tobacco smoking and breast cancer risk,^7^ although they are based on a larger number of studies and included also dose-risk analyses. The dose-response analysis showed a direct linear association with smoking intensity and smoking duration, thus supporting a causal effect of tobacco on breast cancer risk. Similar risk estimates were found for current, former, and ever smokers, and no dose-response association emerged between time since quitting smoking and breast cancer risk.

We found consistent results across strata of various study and population characteristics. In particular, the magnitude of the risk was similar across study designs for former and ever smokers, although it was significantly higher in cohort than in case-control studies for current smokers. Significant heterogeneity in risk estimates were found across geographic areas for ever smokers, with higher associations in the Asian population, but not for current and former smokers. This difference, if not due to chance alone, can be at least partly due to genetic and other differences in this population. Heterogeneity between studies on incidence or mortality was also observed for current smokers, which can be possibly explained by the fact that there may be genetic differences between women who develop breast cancer and those who die from this neoplasm.

We found a higher risk estimate in post-menopausal than in pre-menopausal women among current smokers, but not in former and ever smokers. No difference was found in the previous meta-analysis by Macacu and colleagues based on a more limited number of studies.^7^ Moreover, the stronger risk in post-menopausal women has not a clear explanation, because, if any, the anti-estrogenic effect of tobacco should be stronger in post-menopausal than in premenopausal women.^47^

We did not find evidence of a difference in risk for women with positive and negative ER status. Although in 2014 the report of the Surgeon General of the United States raised the possibility of differences in the risk associated with smoking by hormone receptor status,^32^ results from about 16 studies do not support such a hypothesis.

In our study, we also investigated the possible differential role of tobacco smoking on breast cancer according to different breast cancer susceptibility genes, which have not been considered in previous meta-analyses. Although a few papers suggested a possible stronger effect in BRCA mutation carriers,^8^^–^^10^ our data do not seem to support this hypothesis. Similarly, we found consistent risks according to P53 expression. We observed a somewhat stronger risk in women with NAT2 slow genotype, providing some evidence that the ability to detoxify tobacco carcinogens may modify the risk of tobacco exposure for breast cancer. However, we did not find a stronger risk estimate in women with GSTM1 null genotype, although data on this polymorphism were extremely limited.

Various mechanisms explaining the increased risk of breast cancer in relation to tobacco smoking have been postulated. Tobacco smoke contains several chemical substances, such as polycyclic hydrocarbons, nitrosamines, and aromatic amines, which are known carcinogens.^6^ Specifically, it includes fat-soluble compounds known to induce mammary tumors in rodents.^32^^,^^48^ Since nicotine and other components of cigarette smoke have been found in breast tissue,^49^ these carcinogens are able to reach the breast. Tobacco carcinogens seem, therefore, to overcome the antiestrogenic effect of tobacco, which in the past has been thought to favorably affect breast cancer risk.^4^

The limitations of this work are those typical of meta-analyses of epidemiological studies. Case-control and cohort studies are prone to selection and recall bias. A misclassification of exposure may have occurred since information on smoking intensity and duration was self-reported in most studies. Some misclassification may partly explain the lower association in current than in former smoking observed in case-control studies. Heterogeneity between studies was found for each smoking variable, which may be the result of pooling data from studies conducted with different methodologies, considering different definitions of smoking, and including individuals with various characteristics and background risks. To allow for heterogeneity, we used random-effects models, which give more conservative estimates, although they do not resolve heterogeneity. We also investigated possible putative sources of heterogeneity in risk estimates through stratified analyses according to study, population, and cancer characteristics, although we could not stratify our results for other potentially relevant variables, such as age. However, these variables did not contribute so much to explain the observed heterogeneity.

Our meta-analysis has several strengths. The innovative methodology used to identify original articles—based on a combination of umbrella and traditional reviews^13^^,^^14^—allowed us to include data from 211 epidemiological studies investigating the association between cigarette smoking and breast cancer risk, making this meta-analysis the most comprehensive on the issue. Furthermore, we considered information from publications not indexed in PubMed^5^^,^^6^^,^^31^^–^^33^ that were not captured in previous meta-analyses, although the publications satisfied their inclusion criteria. A careful screening process of the retrieved publications was carried out in order to avoid data overlapping from original studies included in pooled analyses and/or individual-patient meta-analyses. In addition, we modelled the risk functions to describe the best dose-response relationships with smoking intensity, duration, and time since quitting smoking.

In conclusion, this meta-analysis convincingly supports the contention that cigarette smoking is a risk factor for breast cancer. Although the excess risk of breast cancer due to tobacco smoking is of smaller magnitude compared to those of other tobacco-related neoplasms, its impact at a population level may be of great relevance, considering the high incidence of this neoplasm.

## SUPPLEMENTARY MATERIAL

eTable 1. List of 43 eligible publications excluded from the meta-analysis and corresponding reason for exclusion

eTable 2. Main characteristics of the 115 case-control studies on the association between cigarette smoking and breast cancer risk included in the review, and corresponding information contributing to the meta-analysis

eTable 3. Main characteristics of the 54 cohort studies on the association between cigarette smoking and breast cancer risk included in the review, and corresponding information contributing to the meta-analysis

eTable 4. List of publications for which data have been partially excluded from the meta-analysis and reason for exclusion

eFigure 1. Flowchart for the selection of the original studies on the association between cigarette smoking and breast cancer risk included in the review and meta-analysis

eFigure 2. Forest plot of study-specific and pooled relative risk (RR) of breast cancer for ever cigarette smokers (ES) versus never smokers (NS)

eFigure 3. Forest plot of study-specific and pooled relative risk (RR) of breast cancer for ever cigarette smokers (ES) versus never smokers (NS) in women with selected genetic factors

eFigure 4. Funnel plot of studies on the association between current (panel A), former (panel B), and ever (panel C) cigarette smokers versus never smokers and breast cancer risk

eMaterial 1. Literature search strings for the update of the last available comprehensive review used in PubMed/MEDLINE and Embase

eMaterial 2. Functions of the linear models used to estimate the associations between smoking intensity (current vs never smokers), duration (current vs never smokers) and time since quitting (former vs current smokers) and the risk of breast cancer

## References

[1] Sung H, Ferlay J, Siegel RL, . Global Cancer Statistics 2020: GLOBOCAN Estimates of Incidence and Mortality Worldwide for 36 Cancers in 185 Countries. CA Cancer J Clin. 2021;71:209–249. 10.3322/caac.21660 33538338

[2] Ferlay J, Ervik M, Lam F, et al. Global Cancer Observatory: Cancer Today. Lyon, France: International Agency for Research on Cancer; Available from: https://gco.iarc.fr/today, accessed [24/03/2022]. 2020.

[3] Boffetta P, La Vecchia C. Neoplasms. In: Detels R, Beaglehole R, Lansang M, editors. *Oxford Textbook of Public Health 5th Edition*. New York: Oxford University Press; 2009:997–1020.

[4] IARC. Tobacco smoking. Vol 38. IARC Monogr Eval Carcinog Risks Hum. 1986.

[5] IARC Working Group on the Evaluation of Carcinogenic Risks to Humans. Tobacco smoke and involuntary smoking. IARC Monogr Eval Carcinog Risks Hum. 2004;83:1–1438. 15285078 PMC4781536

[6] IARC Working Group on the Evaluation of Carcinogenic Risks to Humans. Personal habits and indoor combustions. IARC Monogr Eval Carcinog Risks Hum. 2012;100(Pt E):1–538. 23193840 PMC4781577

[7] Macacu A, Autier P, Boniol M, Boyle P. Active and passive smoking and risk of breast cancer: a meta-analysis. Breast Cancer Res Treat. 2015;154:213–224. 10.1007/s10549-015-3628-4 26546245

[8] Ginsburg O, Ghadirian P, Lubinski J, ; Hereditary Breast Cancer Clinical Study Group. Smoking and the risk of breast cancer in BRCA1 and BRCA2 carriers: an update. Breast Cancer Res Treat. 2009;114(1):127–135. 10.1007/s10549-008-9977-5 18483851 PMC3033012

[9] Ko KP, Kim SJ, Huzarski T, ; Hereditary Breast Cancer Clinical Study Group. The association between smoking and cancer incidence in BRCA1 and BRCA2 mutation carriers. Int J Cancer. 2018;142(11):2263–2272. 10.1002/ijc.31257 29330845 PMC6020833

[10] Li H, Terry MB, Antoniou AC, . Alcohol consumption, cigarette smoking, and risk of breast cancer for BRCA1 and BRCA2 mutation carriers: results from the BRCA1 and BRCA2 Cohort Consortium. Cancer Epidemiol Biomarkers Prev. 2020;29:368–378. 10.1158/1055-9965.EPI-19-0546 31792088 PMC7611162

[11] Andonova IE, Justenhoven C, Winter S, . No evidence for glutathione S-transferases GSTA2, GSTM2, GSTO1, GSTO2, and GSTZ1 in breast cancer risk. Breast Cancer Res Treat. 2010;121:497–502. 10.1007/s10549-009-0589-5 19859803

[12] van der Hel OL, Peeters PH, Hein DW, . NAT2 slow acetylation and GSTM1 null genotypes may increase postmenopausal breast cancer risk in long-term smoking women. Pharmacogenetics. 2003;13:399–407. 10.1097/00008571-200307000-00005 12835615

[13] Lugo A, Bosetti C, Peveri G, Rota M, Bagnardi V, Gallus S. Dose-response relationship between cigarette smoking and site-specific cancer risk: protocol for a systematic review with an original design combining umbrella and traditional reviews. BMJ Open. 2017;7:e018930. 10.1136/bmjopen-2017-018930 29092902 PMC5695313

[14] Lugo A, Peveri G, Bosetti C, . Strong excess risk of pancreatic cancer for low frequency and duration of cigarette smoking: a comprehensive review and meta-analysis. Eur J Cancer. 2018;104:117–126. 10.1016/j.ejca.2018.09.007 30347287

[15] Santucci C, Bosetti C, Peveri G, . Dose-risk relationships between cigarette smoking and ovarian cancer histotypes: a comprehensive meta-analysis. Cancer Causes Control. 2019;30:1023–1032. 10.1007/s10552-019-01198-8 31236793

[16] Lugo A, Peveri G, Gallus S. Should we consider gallbladder cancer a new smoking-related cancer? A comprehensive meta-analysis focused on dose-response relationships. Int J Cancer. 2020;146:3304–3311. 10.1002/ijc.32681 31513278

[17] Liu X, Peveri G, Bosetti C, . Dose-response relationships between cigarette smoking and kidney cancer: a systematic review and meta-analysis. Crit Rev Oncol Hematol. 2019;142:86–93. 10.1016/j.critrevonc.2019.07.019 31387065

[18] Botteri E, Borroni E, Sloan EK, . Smoking and colorectal cancer risk, overall and by molecular subtypes: a meta-analysis. Am J Gastroenterol. 2020;115:1940–1949. 10.14309/ajg.0000000000000803 32773458

[19] Chen C, Huang YB, Liu XO, . Active and passive smoking with breast cancer risk for Chinese females: a systematic review and meta-analysis. Chin J Cancer. 2014;33:306–316. 10.5732/cjc.013.10248 24823992 PMC4059868

[20] Gaudet MM, Gapstur SM, Sun J, Diver WR, Hannan LM, Thun MJ. Active smoking and breast cancer risk: original cohort data and meta-analysis. J Natl Cancer Inst. 2013;105:515–525. 10.1093/jnci/djt023 23449445

[21] DeRoo LA, Cummings P, Mueller BA. Smoking before the first pregnancy and the risk of breast cancer: a meta-analysis. Am J Epidemiol. 2011;174:390–402. 10.1093/aje/kwr090 21719745 PMC3202162

[22] Nakamura K, Huxley R, Ansary-Moghaddam A, Woodward M. The hazards and benefits associated with smoking and smoking cessation in Asia: a meta-analysis of prospective studies. Tob Control. 2009;18:345–353. 10.1136/tc.2008.028795 19617218

[23] Sadri G, Mahjub H. Passive or active smoking, which is more relevant to breast cancer. Saudi Med J. 2007;28:254–258. 17268706

[24] Nagata C, Mizoue T, Tanaka K, ; Research Group for the Development and Evaluation of Cancer Prevention Strategies in Japan. Tobacco smoking and breast cancer risk: an evaluation based on a systematic review of epidemiological evidence among the Japanese population. Jpn J Clin Oncol. 2006;36(6):387–394. 10.1093/jjco/hyl031 16766567

[25] Lawlor DA, Ebrahim S, Smith GD. Smoking before the birth of a first child is not associated with increased risk of breast cancer: findings from the British Women’s Heart and Health Cohort Study and a meta-analysis. Br J Cancer. 2004;91:512–518. 10.1038/sj.bjc.6601916 15226777 PMC2409831

[26] Gaudet MM, Carter BD, Brinton LA, . Pooled analysis of active cigarette smoking and invasive breast cancer risk in 14 cohort studies. Int J Epidemiol. 2017;46:881–893. 10.1093/ije/dyw288 28031315 PMC5837778

[27] Ordóñez-Mena JM, Schöttker B, Mons U, ; Consortium on Health and Ageing: Network of Cohorts in Europe and the United States (CHANCES). Quantification of the smoking-associated cancer risk with rate advancement periods: meta-analysis of individual participant data from cohorts of the CHANCES consortium. BMC Med. 2016;14:62. 10.1186/s12916-016-0607-5 27044418 PMC4820956

[28] Zheng W, McLerran DF, Rolland BA, . Burden of total and cause-specific mortality related to tobacco smoking among adults aged ≥ 45 years in Asia: a pooled analysis of 21 cohorts. PLoS Med. 2014;11:e1001631. 10.1371/journal.pmed.1001631 24756146 PMC3995657

[29] Cox DG, Dostal L, Hunter DJ, ; Breast and Prostate Cancer Cohort Consortium. N-acetyltransferase 2 polymorphisms, tobacco smoking, and breast cancer risk in the breast and prostate cancer cohort consortium. Am J Epidemiol. 2011;174(11):1316–1322. 10.1093/aje/kwr257 22074863 PMC3390163

[30] Hamajima N, Hirose K, Tajima K, ; Collaborative Group on Hormonal Factors in Breast Cancer. Alcohol, tobacco and breast cancer – collaborative reanalysis of individual data from 53 epidemiological studies, including 58,515 women with breast cancer and 95,067 women without the disease. Br J Cancer. 2002;87:1234–1245. 10.1038/sj.bjc.6600596 12439712 PMC2562507

[31] U.S. Department of Health and Human Services. *The Health Consequences of Smoking: A Report of the Surgeon General*. Atlanta, GA: Centers for Disease Control and Prevention, National Center for Chronic Disease Prevention and Health Promotion, Office on Smoking and Health; 2004.

[32] U.S. Department of Health and Human Services. *The Health Consequences of Smoking—50 Years of Progress. A Report of the Surgeon General*. Rockville, MD: Public Health Service, Office of the Surgeon General; 2014.

[33] U.S. Department of Health and Human Services. *Women and Smoking: A Report of the Surgeon General*. 2001.

[34] Collishaw NB, NF, Cantor KP, Hammond SK, Johnson KC. *Canadian Expert Panel on Tobacco Smoke and Breast Cancer Risk*. Toronto: Ontario Tobacco Research Unit; 2009.

[35] Johnson KC, Miller AB, Collishaw NE, . Active smoking and secondhand smoke increase breast cancer risk: the report of the Canadian Expert Panel on Tobacco Smoke and Breast Cancer Risk (2009). Tob Control. 2011;20:e2. 10.1136/tc.2010.035931 21148114

[36] Hamling J, Lee P, Weitkunat R, Ambühl M. Facilitating meta-analyses by deriving relative effect and precision estimates for alternative comparisons from a set of estimates presented by exposure level or disease category. Stat Med. 2008;27(7):954–970. 10.1002/sim.3013 17676579

[37] Rücker G, Cates CJ, Schwarzer G. Methods for including information from multi-arm trials in pairwise meta-analysis. Res Synth Methods. 2017;8(4):392–403. 10.1002/jrsm.1259 28759708

[38] DerSimonian R, Laird N. Meta-analysis in clinical trials. Control Clin Trials. 1986;7:177–188. 10.1016/0197-2456(86)90046-2 3802833

[39] Higgins JP, Thompson SG. Quantifying heterogeneity in a meta-analysis. Stat Med. 2002;21:1539–1558. 10.1002/sim.1186 12111919

[40] Peters JL, Sutton AJ, Jones DR, Abrams KR, Rushton L. Contour-enhanced meta-analysis funnel plots help distinguish publication bias from other causes of asymmetry. J Clin Epidemiol. 2008;61:991–996. 10.1016/j.jclinepi.2007.11.010 18538991

[41] Egger M, Davey Smith G, Schneider M, Minder C. Bias in meta-analysis detected by a simple, graphical test. BMJ. 1997;315:629–634. 10.1136/bmj.315.7109.629 9310563 PMC2127453

[42] Crippa A, Discacciati A, Bottai M, Spiegelman D, Orsini N. One-stage dose-response meta-analysis for aggregated data. Stat Methods Med Res. 2019;28:1579–1596. 10.1177/0962280218773122 29742975

[43] Desquilbet L, Mariotti F. Dose-response analyses using restricted cubic spline functions in public health research. Stat Med. 2010;29:1037–1057. 10.1002/sim.3841 20087875

[44] Bagnardi V, Rota M, Botteri E, . Alcohol consumption and site-specific cancer risk: a comprehensive dose-response meta-analysis. Br J Cancer. 2015;112:580–593. 10.1038/bjc.2014.579 25422909 PMC4453639

[45] Berlin JA, Longnecker MP, Greenland S. Meta-analysis of epidemiologic dose-response data. Epidemiology. 1993;4:218–228. 10.1097/00001648-199305000-00005 8512986

[46] Crippa AON. Multivariate dose-response meta-analysis: the dosresmeta R package. J Stat Softw. 2016;72.

[47] van den Brandt PA. A possible dual effect of cigarette smoking on the risk of postmenopausal breast cancer. Eur J Epidemiol. 2017;32:683–690. 10.1007/s10654-017-0282-7 28710542 PMC5591344

[48] Phillips DHM, FL, Grover PL, Williams JA. Toxicological basis for a possible association of breast cancer with smoking and other sources of environmental carcinogens. J Women’s Cancer. 2001;3:9–16.

[49] Thompson PA, DeMarini DM, Kadlubar FF, . Evidence for the presence of mutagenic arylamines in human breast milk and DNA adducts in exfoliated breast ductal epithelial cells. Environ Mol Mutagen. 2002;39:134–142. 10.1002/em.10067 11921181

