# Supplementary material for: Dose-response Relationships Between Cigarette Smoking and Breast Cancer Risk: A Systematic Review and Meta-analysis
Source: J Epidemiol. 2023 Dec 5;33(12):640–8. doi: 10.2188/jea.JE20220206 (PMC10635814; doi:10.2188/jea.JE20220206)
Supplement: Supplementary file 1 [file je-33-640-s001.pdf]

Description of Tables or Figure	Page
**eTable 1.** List of 43 eligible publications excluded from the meta-analysis and corresponding reason for exclusion.	2
**eTable 2.** Main characteristics of the 115 case-control studies on the association between cigarette smoking and breast cancer risk included in the review, and corresponding information contributing to the meta-analysis.	4
**eTable 3.** Main characteristics of the 54 cohort studies on the association between cigarette smoking and breast cancer risk included in the review, and corresponding information contributing to the meta-analysis.	10
**eTable 4.** List of publications for which data have been partially excluded from the meta-analysis and reason for exclusion.	14
**eFigure 1.** Flowchart for the selection of the original studies on the association between cigarette smoking and breast cancer risk included in the review and meta-analysis.	15
**eFigure 2**. Forest plot of study-specific and pooled relative risk (RR) of breast cancer for ever cigarette smokers (ES) versus never smokers (NS).	16
**eFigure 3.** Forest plot of study-specific and pooled relative risk (RR) of breast cancer for ever cigarette smokers (ES) versus never smokers (NS) in women with selected genetic factors.	18
**eFigure 4**. Funnel plot of studies on the association between current (panel A), former (panel B), and ever (panel C) cigarette smokers versus never smokers and breast cancer risk.	19
**eMaterial 1.** Literature search strings for the update of the last available comprehensive review used in PubMed/MEDLINE and Embase.	20
**eMaterial 2.** Functions of the linear models used to estimate the associations between smoking intensity (current vs. never smokers), duration (current vs. never smokers) and time since quitting (former vs. current smokers) and the risk of breast cancer.	21
**References**	22

eTable 1. List of 43 eligible publications excluded from the meta-analysis and corresponding reason for exclusion

**First Author, year**	**Study design**	**Reason of exclusion**
Akinyemiju, 2017 [1]	CO	Included in Ordonez-Mena, 2016 [2]
Ambrosone, 1996 [3]	CC	Included in Ambrosone, 2008 [4]
Bjerkaas, 2015 [5]	CO	Included in Bjerkaas, 2013 [6]
Breast Cancer Family, 2008 [7]	CC	Included in Li, 2020 [8] and Zeinomar, 2019 [9]
Britton, 2002 [10]	CC	Included in Gammon, 1998 [11]
Butler, 2016 [12]	CC	Included in Mechanic, 2006 [13]
Chang-Claude, 2002 [14]	CC	Included in Ambrosone, 2008 [4]
Cox, 2011 [15]	PCC	Singles cohorts already included with more information available
Cui, 2006 [16]	CO	Included in Catsburg, 2015 [17]
Dianatinasab, 2017 [18]	CC	Included in Dianatinasab, 2019 [19]
Duell, 2001 [20]	CC	Included in Mechanic, 2006 [13]
Egan, 2002 [21]	CO	Included in Xue, 2011 [22]
Egan, 2003 [23]	CC	Included in Ambrosone, 2008 [4]
Ewertz, 1993 [24]	CC	Included in Ewertz, 1990 [25]
Fararouei, 2019 [26]	CC	Included in Dianatinasab, 2019 [19]
Garcia-Closas, 1999 [27]	NCC	Included in Xue, 2011 [22]
Ghadirian, 2004 [28]	CC	Included in Ginsburg, 2009 [29]
Gram, 2016 [30]	CO	Included in Gaudet, 2017 [31]
Heberg, 2019 [32]	CO	Included in Andersen, 2017 [33]
Ishibe, 1998 [34]	NCC	Included in Xue, 2011 [22]
Key, 2009 [35]	CO	Included in Ordonez-Mena, 2016 [2] and Dossus, 2014 [36]
Lemogne, 2013 [37]	CO	Included in Lemogne, 2013 [38]
Li, 2004 [39]	CC	Included in Mechanic, 2006 [13]
Marcus, 2000 [40]	CC	Included in Mechanic, 2006 [13]
McKenzie, 2015 [41]	CO	Included in Ordonez-Mena, 2016 [2] and Dossus, 2014 [36]
Metsola, 2005 [42]	CC	Included in Sillanpaa, 2005 [43]
Millikan, 1998 [44]	CC	Included in Ambrosone, 2008 [4]
Millikan, 2004 [45]	CC	Included in Mechanic, 2006 [13]
Mitrunen, 2001 [46]	CC	Included in Sillanpaa, 2005 [43]
Mullooly, 2017 [47]	CO	Included in Ordonez-Mena, 2016 [2]
Pachkowski, 2006 [48]	CC	Included in Mechanic, 2006 [13]
Park, 2016 [49]	PCC	Singles cohorts already included with more information available
Schechter, 1985 [50]	CC	Included in Catsburg, 2015 [17]
Schechter, 1989 [51]	CC	Included in Catsburg, 2015 [17]
Shankar, 2008 [52]	CO	Included in Gaudet, 2017 [31]
Shen, 2005 [53]	CC	Included in Mordukhovich, 2010 [54]
Shen, 2005 [55]	CC	Included in Mordukhovich, 2010 [54]

First Author, year	Study design	Reason of exclusion
Shen, 2006 [56]	CC	Included in Mordukhovich, 2010 [54]
Tamimi, 2004 [57]	NCC	Included in Xue, 2011 [22]
Terry, 2002 [58]	CO	Included in Catsburg, 2015 [17]
Van Ryswyk, 2016 [59]	CC	Included in Johnson, 2000 [60]
White, 2016 [61]	CC	Included in Mordukhovich, 2010 [54]
Williams, 2019 [62]	CC	Included in Mechanic, 2006 [13]

CC, case-control study; CO, cohort study; CI, confidence interval; NCC, nested case-control study; NOS, non-original study; PCC, pooled case-control study; RR, relative-risk.

eTable 2. Main characteristics of the 115 case-control studies on the association between cigarette smoking and breast cancer risk included in the review, and corresponding information contributing to the meta-analysis

First author, year	Country	Type of controls	Number of Cases	Number of Controls	Cigarette smoking								Genetic factor	Strata
					Status			Intensity		Duration		TSQ		
					Current	Former	Ever	Current	Ever	Current	Ever	Former		
Adami, 1988 [63]	Sweden, Norway	P	422	527			X		X		X			
Ahern, 2009 [64]	USA	P	557	432										Menop
Alberg, 2004 [65]	USA	P	110	113	X	X	O						NAT2	
Alim, 2016 [66]	Turkey	P	40	40			X							
Alsolami, 2019 [67]	Saudi Arabia	P	214	218			X							
Ambrosone, 2008 [4]	USA, Europe[a]	H/P	5,201	5,829			O				O		NAT2	
Andonova, 2010 [68]	Germany	P	1,143	1,155	X	X	O							
Band, 2002 [69]	Canada	P	1,018	1,025			O		O		O			Menop
Bano, 2016 [70]	Pakistan	P	1,238	1,008			X							
Baron, 1986 [71]	USA	H	1,741	2,128			O							
Baron, 1996 [72]	USA	P	6,888	9,529	X	X	O		X		X	O		Menop
Bennicke, 1995 [73]	Denmark	H	230	3,010			O				X			
Berrandou, 2019 [74]	France	P	1,125	1,172	X	X	O		X		X	X		Menop
Bowlin, 1997 [75]	USA	P	1,214	1,214			X							
Braga, 1996 [76]	Italy	H	2,569	2,588	X	X	X		X		X	O		Menop
Brinton, 1986 [77]	USA	P	1,547	1,930	X	X	X		X		X			Menop
Brown, 2010 [78]	USA	P	597	966	X	X	X		X		X	O		
Brownson, 1988 [79]	USA	P	456	1,693	X	X	O		O		O			
Brunet, 1998 [80]	USA, Canada	H	186	186			X		X				BRCA	
Catsburg, 2014 [81]	Canada	P	1,096	3,314	X	X	X		X		X			Menop

First author, year	Country	Type of controls	Number of Cases	Number of Controls	Cigarette smoking								Genetic factor	Strata
					Status			Intensity		Duration		TSQ		
					Current	Former	Ever	Current	Ever	Current	Ever	Former		
Cerne, 2011 [82]	Slovenia	H	784	709			O		X					
Chu, 1990 [83]	USA	P	4,720	4,682	X	X	X		X		X			Menop
Colilla, 2006 [84]	USA, Canada	H	176	140			X						BRCA1	
Conlon, 2010 [85]	Canada	P	347	775	X	X	X		X		X	O	NAT2	
Connor, 2016 [86]	USA, Mexico	P	3,780	4,137	X	X	O		X		X			Menop
Cooper, 1989 [87]	Australia	P	451	451										ER
Cotterchio, 2003 [88]	Canada	P	3,746	3,691			O							ER, Menop
Croghan, 2009 [89]	USA	H	1,225	6,872			X							
Delfino, 2000 [90]	USA	H	113	278	X	X	O		X		X		NAT2	
Dianatinasab, 2019 [19]	Iran	H	1,009	1,009			X							
Ellingjord-Dale, 2017 [91]	Norway	P	4,402	24,760				X						ER
Ellingjord-Dale, 2018 [92]	Norway	P	4,402	24,760	X	X	O							ER
Ewertz, 1990 [25]	Denmark	P	1,694	1,705	X	X	O		X		X			Menop
Field, 1992 [93]	USA	P	1,617	1,617			X				X			
Furberg, 2002 [94]	USA	P	683	790									p53	
Gammon, 1998 [11]	USA	P	2,199	2,009	X	X	X	X		X		O		ER
Gammon, 1999 [95]	USA	P	509	462									p53	
Gammon, 2004 [96]	USA	P	1,356	1,383										ER, Menop
Gao, 2013 [97]	China	P	669	682			X							
Gaudet, 2005 [98]	USA	P	1,034	1,084				O		O				
Gibson, 2010 [99]	Philippines	P	123	978			X							
Ginsburg, 2009 [29]	Multiple countries	H	2,538	2,538	O	O	X						BRCA	

First author, year	Country	Type of controls	Number of Cases	Number of Controls	Cigarette smoking								Genetic factor	Strata
					Status			Intensity		Duration		TSQ		
					Current	Former	Ever	Current	Ever	Current	Ever	Former		
Godinho-Mota, 2019 [100]	Brazil	P	197	344	X	X	O							
Gronwald, 2006 [101]	Poland	H	348	348			X						BRCA1	
Hamajima, 2002 [102]	Multiple countries	H/P	58,515	95,067	X	X	X							
Hara, 2017 [103]	Japan	H	511	527	X	X	X				X		NAT2	Menop
Hiatt, 1988 [104]	USA	P	303	5,800	O	X	O	X						
Hsieh, 2014 [105]	Taiwan	H/P	737	719			X							
Huang, 1999 [106]	Taiwan	P	150	150			X							
Huang, 2000 [107]	USA	P	862	790										ER, Menop
Hunter, 1997 [108]	USA	P	466	466									NAT2	
Ilic, 2014 [109]	Serbia	H	191	191	X	X	X		X		X	O		
Johnson, 2000 [60]	Canada	P	2,317	2,438	O	O	O							Menop
Kato, 1992 [110]	Japan	H	908	908			X							
Krajinovic, 2001 [111]	Canada	P	149	70			O						NAT2	
Kropp, 2002 [112]	Germany	P	468	1,093	X	X						O		
Kruk, 2007 [113]	Poland	H	858	1,085			O		O					Menop
Lash, 1999 [114]	USA	P	265	765			X		X		X			
Lash, 2002 [115]	USA	P	666	615			X		X		X			
Li, 2005 [116]	USA	P	975	1,005	X	X	X		X		X	O		ER
Li, 2020[b] [8]	Multiple countries[a]	P					X		X				BRCA	
Lilla, 2005 [117]	Germany	P	419	884					O				NAT2	
Lissowska, 2006 [118]	Poland	P	2,386	2,502	X	X			X				NAT2	ER
Magnusson, 2007 [119]	Sweden	P	3,345	3,454	X	X	O	X		X				
Manjer, 2004 [120]	Sweden	P	260	514	X	X	X	X			X	O		

First author, year	Country	Type of controls	Number of Cases	Number of Controls	Cigarette smoking								Genetic factor	Strata
					Status			Intensity		Duration		TSQ		
					Current	Former	Ever	Current	Ever	Current	Ever	Former		
Martin-Moreno, 1993 [121]	Spain	P	762	988			X							
McCredie, 1998 [122]	Australia	P	467	408			X							
McTiernan, 1986 [123]	USA	P	329	332										ER
Meara, 1989 [124]	UK	H/P	1,116	1,116	O	X	O	X						
Mechanic, 2006 [13]	USA	P	2,311	2,022	O	O	O		O		O	O		
Millikan, 2000 [125]	USA	P	688	663									GSTM1	
Morabia, 1996 [126]	Switzerland	P	244	1,032	O	O		X	X					
Morabia, 1998 [127]	Switzerland	P	242	1,059										ER, Menop
Morabia, 2000 [128]	Switzerland	P	177	170									NAT2	
Mordukhovich, 2010 [54]	USA	P	1,508	1,556	O	O	O						p53	
Nishino, 2014 [129]	Japan	H	1,263	3,160	O	O	O		O		O			ER, Menop
O'Connell, 1987 [130]	USA	P	276	1,519	O	X	O	X						
Ozmen, 2009 [131]	Turkey	H	1,492	2,167			X							
Palmer, 1991 [132]	Canada, USA	H/P	2,562	2,019	X	X	X		X					
Pawlega, 1992 [133]	Poland	P	127	250			O				O			
Pieta, 2012 [134]	Poland	H	138	1,144			O							
Pimhanam, 2014 [135]	Thailand	P	444	444			O							
Plagens-Rotman, 2017 [136]	Poland	H	79	683			X		X					
Pongsavee, 2018 [137]	Thailand	P	155	122			X							
Prescott, 2007 [138]	USA	P	1,728	441	X	X	X		X		X			
Rabstein, 2010 [139]	Germany	P	1,020	1,047									NAT2	ER
Rajaei, 2015 [140]	Iran	P	607	438			X							

First author, year	Country	Type of controls	Number of Cases	Number of Controls	Cigarette smoking								Genetic factor	Strata
					Status			Intensity		Duration		TSQ		
					Current	Former	Ever	Current	Ever	Current	Ever	Former		
Ranstam, 1995 [141]	Sweden	P	393	449			O		O					Menop
Rautalahti, 1993 [142]	Finland	P	67	157			X		X		X			Menop
Richardson, 1989 [143]	France	H	349	459			X							
Roddam, 2007 [144]	UK	P	639	640	X	X	O							
Rohan, 1989 [145]	Australia	P	451	451	X	X	O	X						Menop
Rollison, 2008 [146]	USA	P	287	311			X		X		X			
Ruszczyk, 2016 [147]	USA	P	831	1,620			O							
Shore, 2008 [148]	USA	P	612	612			O							
Sillanpaa, 2005 [43]	Finland	P	483	482	X	X							NAT2	
Slattery, 2008 [149]	USA	P	2,556	2,606	O	O	O							Menop
Smith, 1994 [150]	UK	P	755	755			X		X		X			
Stanford, 1987 [151]	USA	P	458	568										ER
Trivers, 2009 [152]	USA	P	831	913	O	O	O							ER
Tung, 1999 [153]	Japan	H	376	430	X	X	O							Menop
Ueji, 1998 [154]	Japan	P	148	303			X							Menop
van der Hel, 2003 [155]	Netherland	P	229	264					X				NAT2, GSTM1	Menop
van der Hel, 2005 [156]	Netherland	P	676	669	O	X		X					NAT2, GSTM1	
Viladiu, 1996 [157]	Spain	P	330	346			X							
Wakai, 1994 [158]	Japan	H	300	900	X	X	O							Menop
Wang, 2011 [159]	China	P	400	400			X							Menop
White, 1994 [160]	USA	P	747	961	X	X	O							

First author, year	Country	Type of controls	Number of Cases	Number of Controls	Cigarette smoking								Genetic factor	Strata
					Status			Intensity		Duration		TSQ		
					Current	Former	Ever	Current	Ever	Current	Ever	Former		
Yang, 1997 [161]	Taiwan	H	224	450			X							Menop
Yoo, 1997 [162]	Japan	H	1,154	21,714			X							ER
Young, 2009 [163]	Canada[a]	P	6,235	6,533			X							
Yuan, 1988 [164]	China	P	534	534			X							
Zheng, 1999 [165]	USA	P	273	657									NAT1	
Zheng, 2002 [166]	USA	H	338	345	X	X	X		X		X		GSTM1	Menop
Zheng, 2011 [167]	China	P	1,541	1,598			X							
Total (1986–2020)			181,415	313,384	46	46	92	11	36	3	31	11	25	40

H, hospital; Menop, Menopausal status; O symbol indicates that estimates were derived from the information provided in the original study publication; P, population; TSQ, time-since-quitting; X symbol indicates that estimates were provided in the original study publication.

[a]Pooled-analysis

[b]This article includes both a case-control study and a cohort study

eTable 3. Main characteristics of the 54 cohort studies on the association between cigarette smoking and breast cancer risk included in the review, and corresponding information contributing to the meta-analysis

First author, year	Country (study acronym)	Endpoint	Number of Cases	Cigarette smoking								Genetic factors	Strata
				Status			Intensity		Duration		TSQ		
				Current	Former	Ever	Current	Ever	Current	Ever	Former		
Akiba, 1990 [168]	Japan (6 prefectures) [a]	m	204	X			X						
Al-Delaimy, 2004 [169]	USA (NHSII)	i	1,009	X	X	O	X			X			ER
Andersen, 2017 [33]	Denmark (DNC)	i	1,162	X	X	X		X		X			ER, Menop
Arthur, 2018 [170]	USA (WHI)	i	8,168	O	O	O	X						
Arthur, 2020 [171]	UK (UK Biobank)	i	3,422	O	O	O							Menop
Bjerkaas, 2013 [6]	Norway (NNHSS)	i	7,490	X	X	X		X		X			
Blakely, 2013 [172]	New Zealand (NZCR)	i	12,384	X	X	O							
Calle, 1994 [173]	USA, Puerto Rico (CPS-II)	m	880	X	X	X	X		X				
Carter, 2015 [174]	USA (multiple cohorts)[b]	m	2,022	X									
Catsburg, 2015 [17]	Canada (NBSS)	i	6,549	X	X	X		X		X	X		
Coleman, 2020 [175]	USA (NHIS)	m	2,099	X	X	O							
Dossus, 2014 [36]	Europe (EPIC)	i	9,822	X	X	X	X		X		X		ER, Menop
Engeland, 1996 [176]	Norway (MS)	i	603	X	X	O							
Gaudet, 2013 [177]	USA (CPS-II)	i	3,721	X	X	O	X		X				ER
Gaudet, 2017 [31]	Multiple countries[b]	i	36,060	X	X	O	X		X				ER, Menop
Goodman, 1997 [178]	Japan (RERF-LSS)	i	161	X	X	X							
Gram, 2005 [179]	Norway, Sweden (NSCS)	i	1,240	X	X	X	X		X				
Gram, 2015 [180]	USA (MEC)	i	4,484	X	X	X		X		X			
Gram, 2019 [181]	USA (MEC)	i	4,230										ER
Hanaoka, 2005 [182]	Japan (JPHC)	i	180	X	X	O							Menop

First author, year	Country (study acronym)	Endpoint	Number of Cases	Cigarette smoking								Genetic factors	Strata
				Status			Intensity		Duration		TSQ		
				Current	Former	Ever	Current	Ever	Current	Ever	Former		
Hiatt, 1986 [183]	USA (KPMCP)	i	1,363	O	X	O	X						Menop
Jacob, 2018 [184]	UK (UKGP)	i	8,924	X									
Jee, 2004 [185]	Korea (KCPS)	m	205	X	X	O							
Jones, 2017 [186]	UK (GSC)	i	1,815	X	X	X		X		X			ER
Ko, 2018 [187]	Multiple countries (MLC_BRCA)	i	428	X	X	X		X		X		BRCA	
Land, 2014 [188]	USA (NSABP P-1)	i	395	X	X	O	X	X	X	X			
Lemogne, 2013 [38]	France (GAZEL)	i	138	O	O	O							
Li, 2020[d] [8]	Multiple countries[b]	i				X		X				BRCA	
Lin, 2008 [189]	Japan (JACC)	i	208	X	X	O	X						
London, 1989 [190]	USA (NHS)	i	1,788										ER, Menop
Luo, 2011 [191]	USA (WHI)	i	3,520							X			ER
Manjer, 2000 [192]	Sweden (DPM)	i	416	X	X	O	X						Menop
Manjer, 2001 [193]	Sweden (DPM)	i	268										ER
Meyer, 2015 [194]	Switzerland (multiple cohorts)[b]	m	204	O	X	O	X						
Nordlund, 1997 [195]	Sweden (SCR)	i	996	X	X	O	X						
Nyante, 2014 [196]	USA (NIH-AARP)	i	7,481										ER
Olson, 2005 [197]	USA (IWHS)	i	2,017	X	X	O				X			
Ordonez-Mena, 2016 [2]	Multiple countries (CHANCES)[b]	i/m	15,085/ 2,568	X	X	O		X		X	X		
Ozasa, 2007 [198]	Japan (JACC)	m	93	X	X		X		X				
Park, 2020 [199]	Korea (NBCSP)	i	55,538			X							Menop
Pirie, 2013 [200]	UK (MWS)	m	3,207	X			X						
Reynolds, 2004 [201]	USA (CTS)	i	2,005	X	X	O		X		X			Menop
Rosenberg, 2013 [202]	USA (BWHS)	i	1,377	X	X	X							Menop

First author, year	Country (study acronym)	Endpoint	Number of Cases	Cigarette smoking								Genetic factors	Strata
				Status			Intensity		Duration		TSQ		
				Current	Former	Ever	Current	Ever	Current	Ever	Former		
Schatzkin, 1989 [203]	USA (FHS)	i	143			O		X					
Swift, 2008 [204]	USA, Canada (ATPF)	i	77			O							
Taghizadeh, 2016 [205]	Netherland (VVS)	m	117	O	X	O	X						
Tverdal, 1993 [206]	Norway (5 areas)[c]	m	70	X	X	O	X						
van den Brandt, 2017 [207]	Netherland (NLCS)	i	2,526	X	X	X		X		X	X		
Viner, 2019 [208]	USA (ATP)	i	483	O	O	O							Menop
Wada, 2015 [209]	Japan (TS)	i	166	X	X	O							
White, 2017 [210]	USA, Puerto Rico (NIEHS)	i	1,843	X	X	X	X		X	X	O		
Xue, 2011 [22]	USA (NHS)	i	8,772	X	X	X	X			X	O		
Zeinomar, 2019 [9]	Multiple countries (ProF-SC)[b]	i	1,009	X	X	O							ER
Zheng, 2014 [211]	Asia (ACC)[b]	m	1,108			O							
Total (1986–2020)			229,675	44	40	44	20	12	8	15	6	2	20

ACC, Asia Cohort Consortium; ATP, Alberta's Tomorrow Project; ATPF, A-T patients and their families; BWHS, Black Women Health Study; CHANCES, Consortium on Health and Aging: Network of Cohorts in Europe and the United States; CPS-II, Cancer Prevention Study II; CTS, California teacher Study; DNC, Danish Nurse Cohort; DPM, Department of Preventive Medicine in Malmö; EPIC, European Prospective Investigation into Cancer and Nutrition cohort study; FHS, Framingham Heart Study; GAZEL, Gaz et Electricité cohort; GSC, Generations Study cohort; i, incidence; IWHS, Iowa Women's Health Study; JACC, Japan Collaborative Cohort Study for Evaluation of Cancer; JPHC, Japan Public Health Center; KCPS, Korean Cancer Prevention Study; KPMCP, Kaiser Permanente Medical Care Program; MEC, Multiethnic Cohort Study; m, mortality; MLC_BRCA, Multicenter longitudinal cohort of BRCA mutation carriers; MS, Multiple sites in the USA and Canada; MWS, Million Women Study; NBCSP, National breast cancer screening program; NBSS, National Breast Screening Study; NHIS, National Health Interview Survey; NHS, Nurses' Health Study; NHSII, Nurses' Health Study II; NIEHS, National Institute of Environmental Health Sciences; NIH-AARP, National Institutes of Health-AARP Diet and Health Study; NLCS, Netherlands Cohort Study; NNHSS, Norwegian National Health Screening Service; NSABP P-1, National Surgical Adjuvant Breast and Bowel Project Breast Cancer Prevention Trial; NSCS, Norwegian Swedish Cohort Study; NZCR, New Zealand Cancer Registry; O symbol indicates that estimates were derived from the information provided in the original study publication; ProF-SC, Prospective Family Study Cohort; RERF-LSS, Radiation Effects Research Foundation's extended Life Span Study; SCR, Swedish Cancer Registry; TS, Takayama Study; UKGP, UK general practitioners; VVS, Vlagtwedde-Vlaardingen study; WHI, Women's Health Initiative; X symbol indicates that estimates were provided in the original study publication.

[a]Six-prefecture Cohort Study
[b]Pooled-analysis
[c]Five areas in Norway
[d]This article includes both a case-control study and a cohort study

eTable 4. List of publications for which data have been partially excluded from the meta-analysis and reason for exclusion

**First author, year**	**Excluded estimate**	**Reason of exclusion**
Ellingjord-Dale, 2017 [91]	Status (current, former)	Included in Ellingjord-Dale, 2018 [92]
Gammon, 2004 [96]	Status (ever)	Included in Mordukhovich, 2010 [54]
Gram, 2019 [181]	Status (current, former, ever), intensity (ever), duration (ever)	Included in Gram, 2015 [180]
Hunter, 1997 [108]	Status (former), intensity (current)	Included in Xue, 2011 [22]
Kropp, 2002 [112]	Duration (ever)	Included in Ambrosone, 2008 [4]
Lissowska, 2006 [118]	Status (ever), duration (ever)	Included in Ambrosone, 2008 [4]
London, 1989 [190]	Status (current), intensity (current)	Included in Xue, 2011 [22]
Luo, 2011 [191]	Status (current, former), intensity (ever)	Included in Arthur, 2018 [170]
Manjer, 2001 [193]	Status (current, former), intensity (current)	Included in Manjer, 2000 [192]
Morabia, 1998 [127]	Intensity (ever)	Included in Morabia, 1996 [126]
Morabia, 2000 [128]	Status (former, ever), intensity (current)	Included in Morabia, 1996 [126] and Ambrosone, 2008 [4]
Nyante, 2014 [196]	Status (current, former), intensity (current, former)	Included in Ordonez-Mena, 2016 [2]
Rabstein, 2010 [139]	Status (current, former)	Included in Andonova, 2010 [68]
van der Hel, 2003 [155]	Duration (ever)	Included in Ambrosone, 2008 [4]
Zheng, 1999 [165]	Status (ever)	Included in Olson, 2005 [197]

eFigure 1. Flowchart for the selection of the original studies on the association between cigarette smoking and breast cancer risk included in the review and meta-analysis

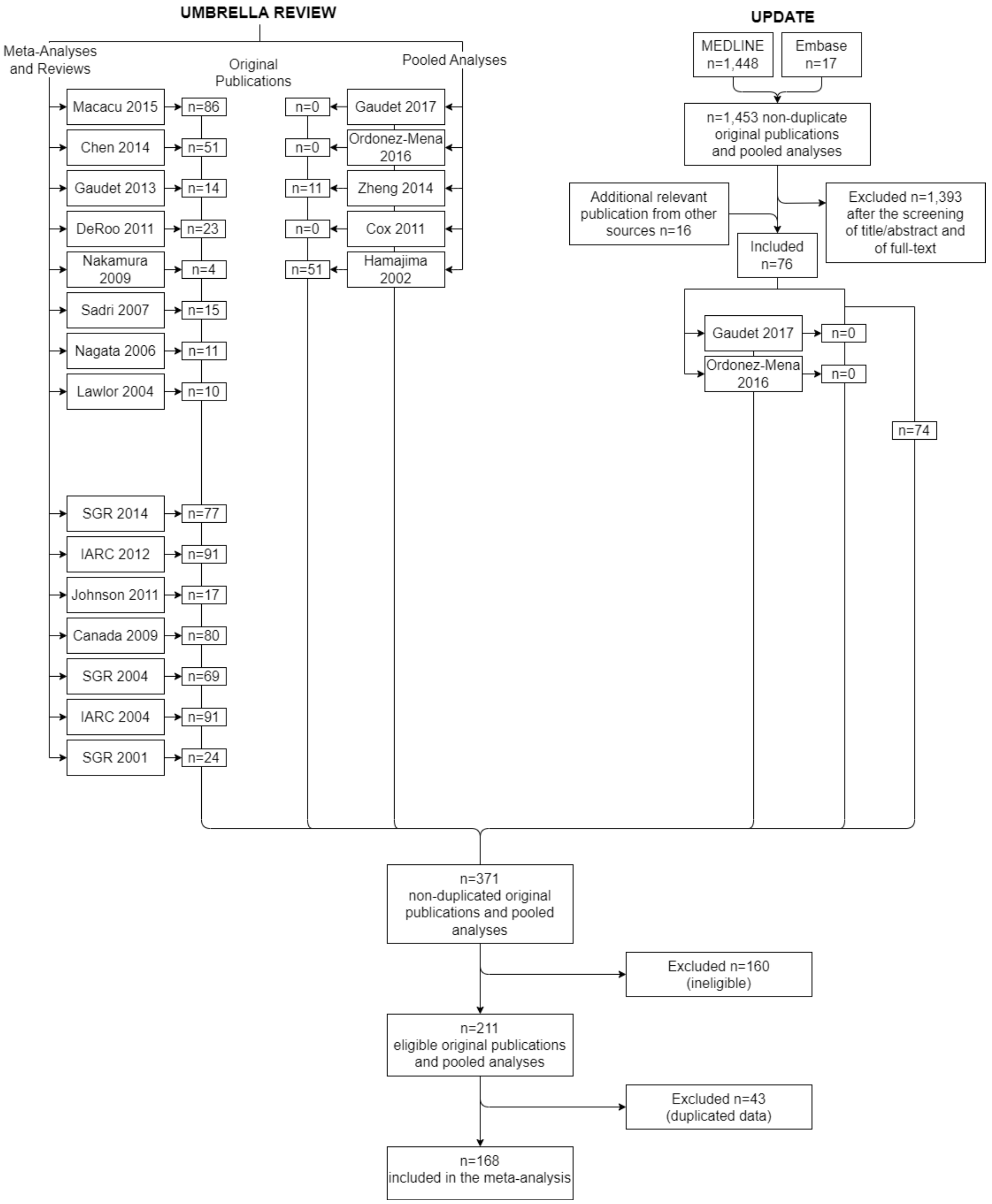

eFigure 2. Forest plot of study-specific and pooled relative risk (RR) of breast cancer for ever cigarette smokers (ES) versus never smokers (NS)

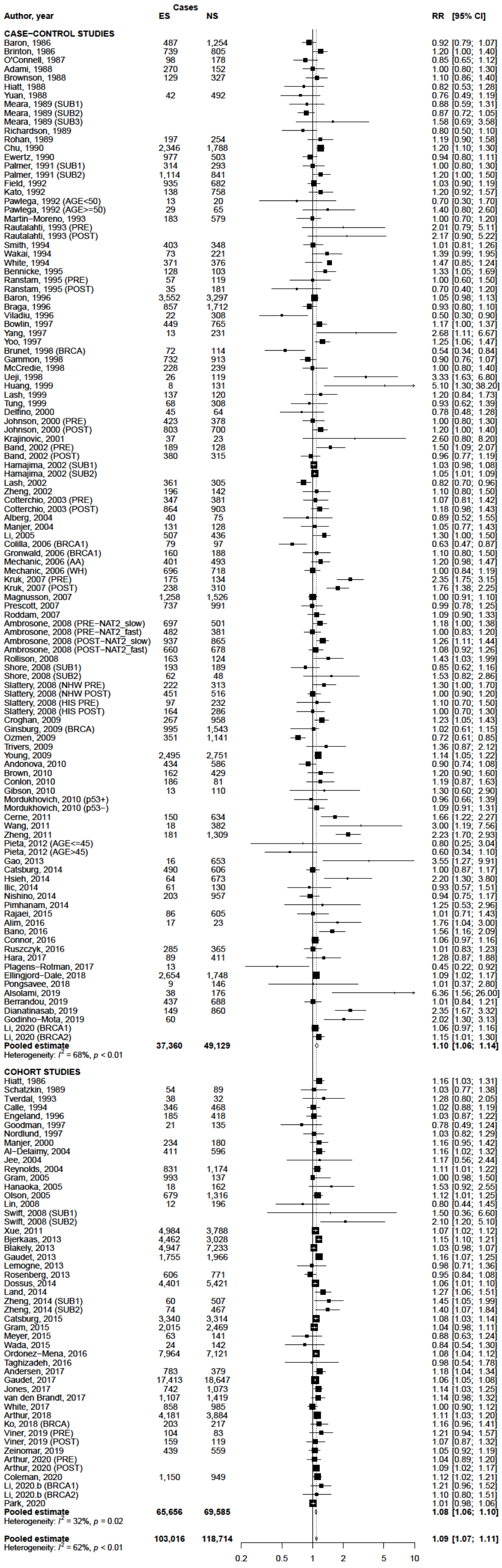

AA, African-American ethnicity; BRCA, BRCA mutation; BRCA1, BRCA1 mutation; BRCA2, BRCA2 mutation; CI, confidence interval; HIS, Hispanic ethnicity; NAT2_fast, NAT2 fast ploymorphism; NAT2_slow, NAT2 slow polymorphism; NHW, non-Hispanic Caucasian ethnicity; p53-, p53 negative; p53+, p53 positive; POST, post-menopausal women; PRE, pre-menopausal women; SUB1, first subgroup; SUB2, second subgroup; SUB3, third subgroup; WH, white ethnicity.

eFigure 3. Forest plot of study-specific and pooled relative risk (RR) of breast cancer for ever cigarette smokers (ES) versus never smokers (NS) in women with selected genetic factors

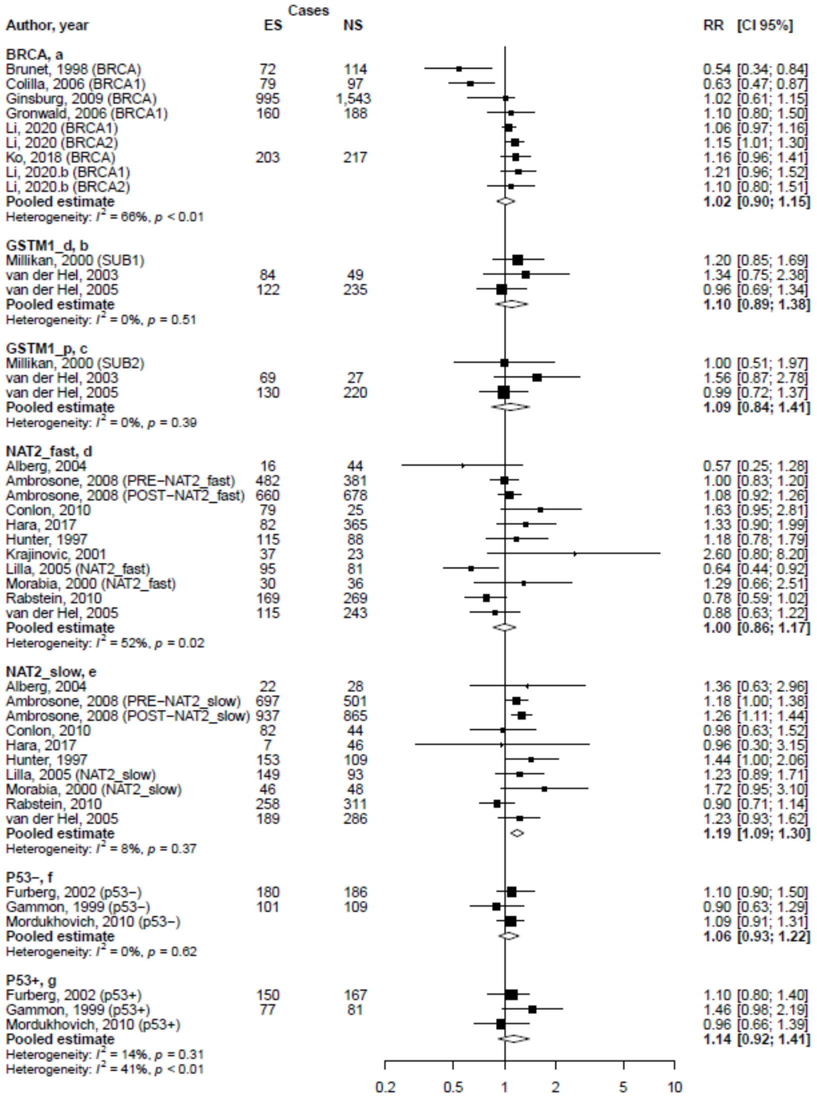

BRCA, BRCA mutation; BRCA1, BRCA1 mutation; BRCA2, BRCA2 mutation; CI, confidence interval; GSTM1_d, GSTM1 null genotype; GSTM1_p, GSTM1 present genotype; NAT2_fast, NAT2 fast ploymorphism; NAT2_slow, NAT2 slow polymorphism; p53-, p53 negative; p53+, p53 positive; POST, post-menopausal women; PRE, pre-menopausal women; SUB1, first subgroup; SUB2, second subgroup.

eFigure 4. Funnel plot of studies on the association between current (panel A), former (panel B), and ever (panel C) cigarette smokers versus never smokers and breast cancer risk

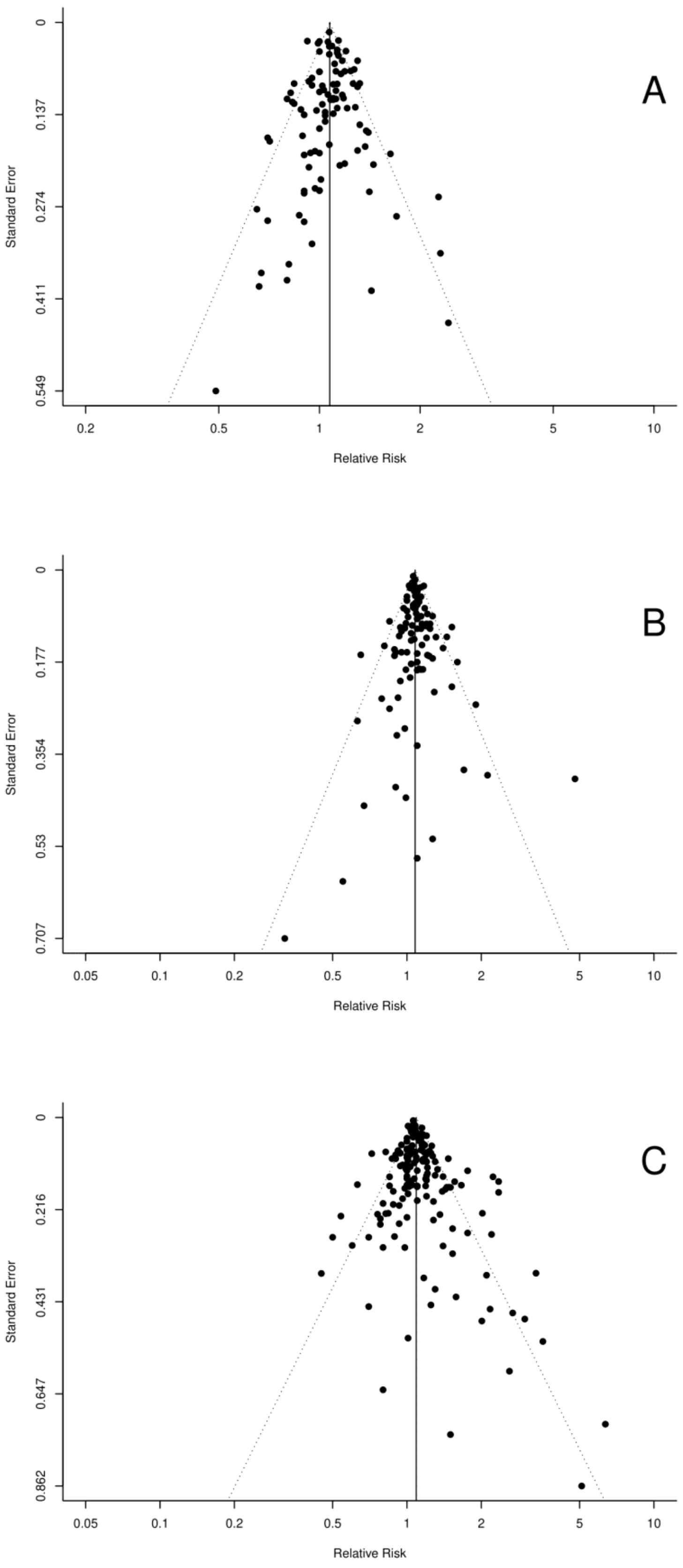

eMaterial 1. Literature search strings for the update of the last available comprehensive review used in PubMed/MEDLINE and Embase

Source	Date	Search string	N
PubMed	9/6/2020	(breast) AND<br>(cancer OR neoplasm OR carcinoma OR adenocarcinoma OR Neoplasms [MeSH Terms]) AND<br>(cigarette OR cigarettes OR tobacco OR smoking OR smokers OR smoking [MeSH Terms]) AND<br>(English[Language]) AND<br>("2015"[Date - Publication]: "2020"[Date - Publication])	1448
Embase	9/6/2020	cigarette:ti OR cigarettes:ti OR tobacco:ti OR smoking:ti OR smokers:ti AND (breast:ab,ti) AND (cancer:ab,ti OR neoplasm:ab,ti OR carcinoma:ab,ti OR adenocarcinoma:ab,ti) AND (article:it OR review:it) AND [english]/lim AND [2015-2020]/py NOT [medline]/lim	17
		Duplicates	12
TOT	9/6/2020	-	1453<br>non duplicates

eMaterial 2. Functions of the linear models used to estimate the associations between smoking intensity (current vs. never smokers), duration (current vs. never smokers) and time since quitting (former vs. current smokers) and the risk of breast cancer

Smoking intensity (cigarettes/day)

$f(x) = 0.0057740x$

Smoking duration (years)

$f(x) = 0.0026293x$

Time since quitting (years)

$f(x) = -0.0013354x$

REFERENCES

1. Akinyemiju T, Wiener H, Pisu M. Cancer-related risk factors and incidence of major cancers by race, gender and region; analysis of the NIH-AARP diet and health study. BMC Cancer 2017; 17: 597.
2. Ordóñez-Mena JM, Schöttker B, Mons U et al.; Consortium on Health and Ageing: Network of Cohorts in Europe and the United States (CHANCES). Quantification of the smoking-associated cancer risk with rate advancement periods: meta-analysis of individual participant data from cohorts of the CHANCES consortium. BMC Med 2016; 14: 62.
3. Ambrosone CB, Freudenheim JL, Graham S et al. Cigarette smoking, N-acetyltransferase 2 genetic polymorphisms, and breast cancer risk. JAMA 1996; 276: 1494-1501.
4. Ambrosone CB, Kropp S, Yang J et al. Cigarette smoking, N-acetyltransferase 2 genotypes, and breast cancer risk: pooled analysis and meta-analysis. Cancer Epidemiol Biomarkers Prev 2008; 17: 15-26.
5. Bjerkaas E, Parajuli R, Engeland A et al. Social inequalities and smoking-associated breast cancer - Results from a prospective cohort study. Prev Med 2015; 73: 125-129.
6. Bjerkaas E, Parajuli R, Weiderpass E et al. Smoking duration before first childbirth: an emerging risk factor for breast cancer? Results from 302,865 Norwegian women. Cancer Causes Control 2013; 24: 1347-1356.
7. Breast Cancer Family Registry; Kathleen Cuningham Consortium for Research into Familial Breast Cancer (Australasia); Ontario Cancer Genetics Network (Canada). Smoking and risk of breast cancer in carriers of mutations in BRCA1 or BRCA2 aged less than 50 years. Breast Cancer Res Treat 2008; 109: 67-75.
8. Li H, Terry MB, Antoniou AC et al. Alcohol Consumption, Cigarette Smoking, and Risk of Breast Cancer for BRCA1 and BRCA2 Mutation Carriers: Results from The BRCA1 and BRCA2 Cohort Consortium. Cancer Epidemiol Biomarkers Prev 2020; 29: 368-378.
9. Zeinomar N, Knight JA, Genkinger JM et al. Alcohol consumption, cigarette smoking, and familial breast cancer risk: findings from the Prospective Family Study Cohort (ProF-SC). Breast Cancer Res 2019; 21: 128.
10. Britton JA, Gammon MD, Schoenberg JB et al. Risk of breast cancer classified by joint estrogen receptor and progesterone receptor status among women 20-44 years of age. Am J Epidemiol 2002; 156: 507-516.
11. Gammon MD, Schoenberg JB, Teitelbaum SL et al. Cigarette smoking and breast cancer risk among young women (United States). Cancer Causes Control 1998; 9: 583-590.
12. Butler EN, Tse CK, Bell ME et al. Active smoking and risk of Luminal and Basal-like breast cancer subtypes in the Carolina Breast Cancer Study. Cancer Causes Control 2016; 27: 775-786.
13. Mechanic LE, Millikan RC, Player J et al. Polymorphisms in nucleotide excision repair genes, smoking and breast cancer in African Americans and whites: a population-based case-control study. Carcinogenesis 2006; 27: 1377-1385.
14. Chang-Claude J, Kropp S, Jäger B et al. Differential effect of NAT2 on the association between active and passive smoke exposure and breast cancer risk. Cancer Epidemiol Biomarkers Prev 2002; 11: 698-704.
15. Cox DG, Dostal L, Hunter DJ et al.; Breast and Prostate Cancer Cohort Consortium. N-acetyltransferase 2 polymorphisms, tobacco smoking, and breast cancer risk in the breast and prostate cancer cohort consortium. Am J Epidemiol 2011; 174: 1316-1322.
16. Cui Y, Miller AB, Rohan TE. Cigarette smoking and breast cancer risk: update of a prospective cohort study. Breast Cancer Res Treat 2006; 100: 293-299.
17. Catsburg C, Miller AB, Rohan TE. Active cigarette smoking and risk of breast cancer. Int J Cancer 2015; 136: 2204-2209.
18. Dianatinasab M, Fararouei M, Mohammadianpanah M et al. Hair Coloring, Stress, and Smoking Increase the Risk of Breast Cancer: A Case-Control Study. Clin Breast Cancer 2017; 17: 650-659.
19. Dianatinasab M, Fararouei M, Daneshi N et al. Heterogeneity in risk factors for ductal and lobular breast carcinomas: A case-control study. Int J Cancer 2019; 145: 2917-2925.
20. Duell EJ, Millikan RC, Pittman GS et al. Polymorphisms in the DNA repair gene XRCC1 and breast cancer. Cancer Epidemiol Biomarkers Prev 2001; 10: 217-222.

21. Egan KM, Stampfer MJ, Hunter D et al.; Nurses' Health Study. Active and passive smoking in breast cancer: prospective results from the Nurses' Health Study. Epidemiology 2002; 13: 138-145.
22. Xue F, Willett WC, Rosner BA et al. Cigarette smoking and the incidence of breast cancer. Arch Intern Med 2011; 171: 125-133.
23. Egan KM, Newcomb PA, Titus-Ernstoff L et al. Association of NAT2 and smoking in relation to breast cancer incidence in a population-based case-control study (United States). Cancer Causes Control 2003; 14: 43-51.
24. Ewertz M. Breast cancer in Denmark. Incidence, risk factors, and characteristics of survival. Acta Oncol 1993; 32: 595-615.
25. Ewertz M. Smoking and breast cancer risk in Denmark. Cancer Causes Control 1990; 1: 31-37.
26. Fararouei M, Iqbal A, Rezaian S et al. Dietary Habits and Physical Activity are Associated With the Risk of Breast Cancer Among Young Iranian Women: A Case-control Study on 1010 Premenopausal Women. Clin Breast Cancer 2019; 19: e127-e134.
27. García-Closas M, Kelsey KT, Hankinson SE et al. Glutathione S-transferase mu and theta polymorphisms and breast cancer susceptibility. J Natl Cancer Inst 1999; 91: 1960-1964.
28. Ghadirian P, Lubinski J, Lynch H et al. Smoking and the risk of breast cancer among carriers of BRCA mutations. Int J Cancer 2004; 110: 413-416.
29. Ginsburg O, Ghadirian P, Lubinski J et al.; Hereditary Breast Cancer Clinical Study Group. Smoking and the risk of breast cancer in BRCA1 and BRCA2 carriers: an update. Breast Cancer Res Treat 2009; 114: 127-135.
30. Gram IT, Little MA, Lund E, Braaten T. The fraction of breast cancer attributable to smoking: The Norwegian women and cancer study 1991-2012. Br J Cancer 2016; 115: 616-623.
31. Gaudet MM, Carter BD, Brinton LA et al. Pooled analysis of active cigarette smoking and invasive breast cancer risk in 14 cohort studies. Int J Epidemiol 2017; 46: 881-893.
32. Heberg J, Simonsen MK, Danielsen AK et al. Joint tobacco smoking and alcohol intake exacerbates cancer risk in women- the Danish nurse cohort. Eur J Oncol Nurs 2019; 43: 101675.
33. Andersen ZJ, Jørgensen JT, Grøn R et al. Active smoking and risk of breast cancer in a Danish nurse cohort study. BMC Cancer 2017; 17: 556.
34. Ishibe N, Hankinson SE, Colditz GA et al. Cigarette smoking, cytochrome P450 1A1 polymorphisms, and breast cancer risk in the Nurses' Health Study. Cancer Res 1998; 58: 667-671.
35. Key TJ, Appleby PN, Spencer EA et al. Cancer incidence in vegetarians: results from the European Prospective Investigation into Cancer and Nutrition (EPIC-Oxford). Am J Clin Nutr 2009; 89: 1620S-1626S.
36. Dossus L, Boutron-Ruault MC, Kaaks R et al. Active and passive cigarette smoking and breast cancer risk: results from the EPIC cohort. Int J Cancer 2014; 134: 1871-1888.
37. Lemogne C, Consoli SM, Geoffroy-Perez B et al. Personality and the risk of cancer: a 16-year follow-up study of the GAZEL cohort. Psychosom Med 2013; 75: 262-271.
38. Lemogne C, Consoli SM, Melchior M et al. Depression and the risk of cancer: a 15-year follow-up study of the GAZEL cohort. Am J Epidemiol 2013; 178: 1712-1720.
39. Li Y, Millikan RC, Bell DA et al. Cigarette smoking, cytochrome P4501A1 polymorphisms, and breast cancer among African-American and white women. Breast Cancer Res 2004; 6: R460-473.
40. Marcus PM, Newman B, Millikan RC et al. The associations of adolescent cigarette smoking, alcoholic beverage consumption, environmental tobacco smoke, and ionizing radiation with subsequent breast cancer risk (United States). Cancer Causes Control 2000; 11: 271-278.
41. McKenzie F, Ferrari P, Freisling H et al. Healthy lifestyle and risk of breast cancer among postmenopausal women in the European Prospective Investigation into Cancer and Nutrition cohort study. Int J Cancer 2015; 136: 2640-2648.
42. Metsola K, Kataja V, Sillanpää P et al. XRCC1 and XPD genetic polymorphisms, smoking and breast cancer risk in a Finnish case-control study. Breast Cancer Res 2005; 7: R987-997.
43. Sillanpää P, Hirvonen A, Kataja V et al. NAT2 slow acetylator genotype as an important modifier of breast cancer risk. Int J Cancer 2005; 114: 579-584.
44. Millikan RC, Pittman GS, Newman B et al. Cigarette smoking, N-acetyltransferases 1 and 2, and breast cancer risk. Cancer Epidemiol Biomarkers Prev 1998; 7: 371-378.

45. Millikan RC, Player J, de Cotret AR et al. Manganese superoxide dismutase Ala-9Val polymorphism and risk of breast cancer in a population-based case-control study of African Americans and whites. Breast Cancer Res 2004; 6: R264-274.
46. Mitrunen K, Jourenkova N, Kataja V et al. Glutathione S-transferase M1, M3, P1, and T1 genetic polymorphisms and susceptibility to breast cancer. Cancer Epidemiol Biomarkers Prev 2001; 10: 229-236.
47. Mullooly M, Khodr ZG, Dallal CM et al. Epidemiologic Risk Factors for In Situ and Invasive Breast Cancers Among Postmenopausal Women in the National Institutes of Health-AARP Diet and Health Study. Am J Epidemiol 2017; 186: 1329-1340.
48. Pachkowski BF, Winkel S, Kubota Y et al. XRCC1 genotype and breast cancer: functional studies and epidemiologic data show interactions between XRCC1 codon 280 His and smoking. Cancer Res 2006; 66: 2860-2868.
49. Park SY, Palmer JR, Rosenberg L et al. A case-control analysis of smoking and breast cancer in African American women: findings from the AMBER Consortium. Carcinogenesis 2016; 37: 607-615.
50. Schechter MT, Miller AB, Howe GR. Cigarette smoking and breast cancer: a case-control study of screening program participants. Am J Epidemiol 1985; 121: 479-487.
51. Schechter MT, Miller AB, Howe GR et al. Cigarette smoking and breast cancer: case-control studies of prevalent and incident cancer in the Canadian National Breast Screening Study. Am J Epidemiol 1989; 130: 213-220.
52. Shankar A, Yuan JM, Koh WP et al. Morbidity and mortality in relation to smoking among women and men of Chinese ethnicity: the Singapore Chinese Health Study. Eur J Cancer 2008; 44: 100-109.
53. Shen J, Gammon MD, Terry MB et al. Polymorphisms in XRCC1 modify the association between polycyclic aromatic hydrocarbon-DNA adducts, cigarette smoking, dietary antioxidants, and breast cancer risk. Cancer Epidemiol Biomarkers Prev 2005; 14: 336-342.
54. Mordukhovich I, Rossner P, Jr., Terry MB et al. Associations between polycyclic aromatic hydrocarbon-related exposures and p53 mutations in breast tumors. Environ Health Perspect 2010; 118: 511-518.
55. Shen J, Terry MB, Gammon MD et al. MGMT genotype modulates the associations between cigarette smoking, dietary antioxidants and breast cancer risk. Carcinogenesis 2005; 26: 2131-2137.
56. Shen J, Terry MB, Gammon MD et al. IGHMBP2 Thr671Ala polymorphism might be a modifier for the effects of cigarette smoking and PAH-DNA adducts to breast cancer risk. Breast Cancer Res Treat 2006; 99: 1-7.
57. Tamimi RM, Hankinson SE, Spiegelman D et al. Manganese superoxide dismutase polymorphism, plasma antioxidants, cigarette smoking, and risk of breast cancer. Cancer Epidemiol Biomarkers Prev 2004; 13: 989-996.
58. Terry PD, Miller AB, Rohan TE. Cigarette smoking and breast cancer risk: a long latency period? Int J Cancer 2002; 100: 723-728.
59. Van Ryswyk K, Villeneuve PJ, Johnson KC.; Canadian Cancer Registries Epidemiology Research Group. Dietary patterns and the risk of female breast cancer among participants of the Canadian National Enhanced Cancer Surveillance System. Can J Public Health 2016; 107: e49-e55.
60. Johnson KC, Hu J, Mao Y, Canadian Cancer Registries Epidemiology Research Group. Passive and active smoking and breast cancer risk in Canada, 1994-97. Cancer Causes Control 2000; 11: 211-221.
61. White AJ, Bradshaw PT, Herring AH et al. Exposure to multiple sources of polycyclic aromatic hydrocarbons and breast cancer incidence. Environ Int 2016; 89-90: 185-192.
62. Williams LA, Casbas-Hernandez P, Nichols HB et al. Risk factors for Luminal A ductal carcinoma in situ (DCIS) and invasive breast cancer in the Carolina Breast Cancer Study. PLoS One 2019; 14: e0211488.
63. Adami HO, Lund E, Bergström R, Meirik O. Cigarette smoking, alcohol consumption and risk of breast cancer in young women. Br J Cancer 1988; 58: 832-837.
64. Ahern TP, Lash TL, Egan KM, Baron JA. Lifetime tobacco smoke exposure and breast cancer incidence. Cancer Causes Control 2009; 20: 1837-1844.

65. Alberg AJ, Daudt A, Huang HY et al. N-acetyltransferase 2 (NAT2) genotypes, cigarette smoking, and the risk of breast cancer. Cancer Detect Prev 2004; 28: 187-193.
66. Alim NE, Kiziltan G. Assessment of Risk Factors of Obesity and Diet on Breast Cancer in Ankara, Turkey. Pak J Med Sci 2016; 32: 1537-1542.
67. Alsolami FJ, Azzeh FS, Ghafouri KJ et al. Determinants of breast cancer in Saudi women from Makkah region: a case-control study (breast cancer risk factors among Saudi women). BMC Public Health 2019; 19: 1554.
68. Andonova IE, Justenhoven C, Winter S et al. No evidence for glutathione S-transferases GSTA2, GSTM2, GSTO1, GSTO2, and GSTZ1 in breast cancer risk. Breast Cancer Res Treat 2010; 121: 497-502.
69. Band PR, Le ND, Fang R, Deschamps M. Carcinogenic and endocrine disrupting effects of cigarette smoke and risk of breast cancer. Lancet 2002; 360: 1044-1049.
70. Bano R, Ismail M, Nadeem A et al. Potential Risk Factors for Breast Cancer in Pakistani Women. Asian Pac J Cancer Prev 2016; 17: 4307-4312.
71. Baron JA, Byers T, Greenberg ER et al. Cigarette smoking in women with cancers of the breast and reproductive organs. J Natl Cancer Inst 1986; 77: 677-680.
72. Baron JA, Newcomb PA, Longnecker MP et al. Cigarette smoking and breast cancer. Cancer Epidemiol Biomarkers Prev 1996; 5: 399-403.
73. Bennicke K, Conrad C, Sabroe S, Sørensen HT. Cigarette smoking and breast cancer. BMJ 1995; 310: 1431-1433.
74. Berrandou T, Mulot C, Cordina-Duverger E et al. Association of breast cancer risk with polymorphisms in genes involved in the metabolism of xenobiotics and interaction with tobacco smoking: A gene-set analysis. Int J Cancer 2019; 144: 1896-1908.
75. Bowlin SJ, Leske MC, Varma A et al. Breast cancer risk and alcohol consumption: results from a large case-control study. Int J Epidemiol 1997; 26: 915-923.
76. Braga C, Negri E, La Vecchia C et al. Cigarette smoking and the risk of breast cancer. Eur J Cancer Prev 1996; 5: 159-164.
77. Brinton LA, Schairer C, Stanford JL, Hoover RN. Cigarette smoking and breast cancer. Am J Epidemiol 1986; 123: 614-622.
78. Brown LM, Gridley G, Wu AH et al. Low level alcohol intake, cigarette smoking and risk of breast cancer in Asian-American women. Breast Cancer Res Treat 2010; 120: 203-210.
79. Brownson RC, Blackwell CW, Pearson DK et al. Risk of breast cancer in relation to cigarette smoking. Arch Intern Med 1988; 148: 140-144.
80. Brunet JS, Ghadirian P, Rebbeck TR et al. Effect of smoking on breast cancer in carriers of mutant BRCA1 or BRCA2 genes. J Natl Cancer Inst 1998; 90: 761-766.
81. Catsburg C, Kirsh VA, Soskolne CL et al. Active cigarette smoking and the risk of breast cancer: a cohort study. Cancer Epidemiol 2014; 38: 376-381.
82. Cerne JZ, Ferk P, Leskosek B, Gersak K. Hormone replacement therapy and some risk factors for breast cancer among Slovenian postmenopausal women. Climacteric 2011; 14: 458-463.
83. Chu SY, Stroup NE, Wingo PA et al. Cigarette smoking and the risk of breast cancer. Am J Epidemiol 1990; 131: 244-253.
84. Colilla S, Kantoff PW, Neuhausen SL et al. The joint effect of smoking and AIB1 on breast cancer risk in BRCA1 mutation carriers. Carcinogenesis 2006; 27: 599-605.
85. Conlon MS, Johnson KC, Bewick MA et al. Smoking (active and passive), N-acetyltransferase 2, and risk of breast cancer. Cancer Epidemiol 2010; 34: 142-149.
86. Connor AE, Baumgartner KB, Baumgartner RN et al. Cigarette Smoking and Breast Cancer Risk in Hispanic and Non-Hispanic White Women: The Breast Cancer Health Disparities Study. J Womens Health (Larchmt) 2016; 25: 299-310.
87. Cooper JA, Rohan TE, Cant EL et al. Risk factors for breast cancer by oestrogen receptor status: a population-based case-control study. Br J Cancer 1989; 59: 119-125.
88. Cotterchio M, Kreiger N, Theis B et al. Hormonal factors and the risk of breast cancer according to estrogen- and progesterone-receptor subgroup. Cancer Epidemiol Biomarkers Prev 2003; 12: 1053-1060.
89. Croghan IT, Pruthi S, Hays JT et al. The role of smoking in breast cancer development: an analysis of a Mayo Clinic cohort. Breast J 2009; 15: 489-495.

90. Delfino RJ, Smith C, West JG et al. Breast cancer, passive and active cigarette smoking and N-acetyltransferase 2 genotype. Pharmacogenetics 2000; 10: 461-469.
91. Ellingjord-Dale M, Vos L, Hjerkind KV et al. Alcohol, Physical Activity, Smoking, and Breast Cancer Subtypes in a Large, Nested Case-Control Study from the Norwegian Breast Cancer Screening Program. Cancer Epidemiol Biomarkers Prev 2017; 26: 1736-1744.
92. Ellingjord-Dale M, Vos L, Vik Hjerkind K et al. Number of Risky Lifestyle Behaviors and Breast Cancer Risk. JNCI Cancer Spectr 2018; 2: pky030.
93. Field NA, Baptiste MS, Nasca PC, Metzger BB. Cigarette smoking and breast cancer. Int J Epidemiol 1992; 21: 842-848.
94. Furberg H, Millikan RC, Geradts J et al. Environmental factors in relation to breast cancer characterized by p53 protein expression. Cancer Epidemiol Biomarkers Prev 2002; 11: 829-835.
95. Gammon MD, Hibshoosh H, Terry MB et al. Cigarette smoking and other risk factors in relation to p53 expression in breast cancer among young women. Cancer Epidemiol Biomarkers Prev 1999; 8: 255-263.
96. Gammon MD, Eng SM, Teitelbaum SL et al. Environmental tobacco smoke and breast cancer incidence. Environ Res 2004; 96: 176-185.
97. Gao CM, Ding JH, Li SP et al. Active and passive smoking, and alcohol drinking and breast cancer risk in chinese women. Asian Pac J Cancer Prev 2013; 14: 993-996.
98. Gaudet MM, Gammon MD, Santella RM et al. MnSOD Val-9Ala genotype, pro- and anti-oxidant environmental modifiers, and breast cancer among women on Long Island, New York. Cancer Causes Control 2005; 16: 1225-1234.
99. Gibson LJ, Héry C, Mitton N et al. Risk factors for breast cancer among Filipino women in Manila. Int J Cancer 2010; 126: 515-521.
100. Godinho-Mota JCM, Gonçalves LV, Mota JF et al. Sedentary Behavior and Alcohol Consumption Increase Breast Cancer Risk Regardless of Menopausal Status: A Case-Control Study. Nutrients 2019; 11.
101. Gronwald J, Byrski T, Huzarski T et al. Influence of selected lifestyle factors on breast and ovarian cancer risk in BRCA1 mutation carriers from Poland. Breast Cancer Res Treat 2006; 95: 105-109.
102. Hamajima N, Hirose K, Tajima K et al.; Collaborative Group on Hormonal Factors in Breast Cancer. Alcohol, tobacco and breast cancer--collaborative reanalysis of individual data from 53 epidemiological studies, including 58,515 women with breast cancer and 95,067 women without the disease. Br J Cancer 2002; 87: 1234-1245.
103. Hara A, Taira N, Mizoo T et al. N-acetyltransferase 2 polymorphism and breast cancer risk with smoking: a case control study in Japanese women. Breast Cancer 2017; 24: 254-262.
104. Hiatt RA, Klatsky AL, Armstrong MA. Alcohol consumption and the risk of breast cancer in a prepaid health plan. Cancer Res 1988; 48: 2284-2287.
105. Hsieh YC, Lee CH, Tu SH et al. CHRNA9 polymorphisms and smoking exposure synergize to increase the risk of breast cancer in Taiwan. Carcinogenesis 2014; 35: 2520-2525.
106. Huang CS, Shen CY, Chang KJ et al. Cytochrome P4501A1 polymorphism as a susceptibility factor for breast cancer in postmenopausal Chinese women in Taiwan. Br J Cancer 1999; 80: 1838-1843.
107. Huang WY, Newman B, Millikan RC et al. Hormone-related factors and risk of breast cancer in relation to estrogen receptor and progesterone receptor status. Am J Epidemiol 2000; 151: 703-714.
108. Hunter DJ, Hankinson SE, Hough H et al. A prospective study of NAT2 acetylation genotype, cigarette smoking, and risk of breast cancer. Carcinogenesis 1997; 18: 2127-2132.
109. Ilic M, Vlajinac H, Marinkovic J. Cigarette smoking and breast cancer: a case-control study in Serbia. Asian Pac J Cancer Prev 2014; 14: 6643-6647.
110. Kato I, Miura S, Kasumi F et al. A case-control study of breast cancer among Japanese women: with special reference to family history and reproductive and dietary factors. Breast Cancer Res Treat 1992; 24: 51-59.
111. Krajinovic M, Ghadirian P, Richer C et al. Genetic susceptibility to breast cancer in French-Canadians: role of carcinogen-metabolizing enzymes and gene-environment interactions. Int J Cancer 2001; 92: 220-225.

112. Kropp S, Chang-Claude J. Active and passive smoking and risk of breast cancer by age 50 years among German women. Am J Epidemiol 2002; 156: 616-626.
113. Kruk J. Association of lifestyle and other risk factors with breast cancer according to menopausal status: a case-control study in the Region of Western Pomerania (Poland). Asian Pac J Cancer Prev 2007; 8: 513-524.
114. Lash TL, Aschengrau A. Active and passive cigarette smoking and the occurrence of breast cancer. Am J Epidemiol 1999; 149: 5-12.
115. Lash TL, Aschengrau A. A null association between active or passive cigarette smoking and breast cancer risk. Breast Cancer Res Treat 2002; 75: 181-184.
116. Li CI, Malone KE, Daling JR. The relationship between various measures of cigarette smoking and risk of breast cancer among older women 65-79 years of age (United States). Cancer Causes Control 2005; 16: 975-985.
117. Lilla C, Risch A, Kropp S, Chang-Claude J. SULT1A1 genotype, active and passive smoking, and breast cancer risk by age 50 years in a German case-control study. Breast Cancer Res 2005; 7: R229-237.
118. Lissowska J, Brinton LA, Zatonski W et al. Tobacco smoking, NAT2 acetylation genotype and breast cancer risk. Int J Cancer 2006; 119: 1961-1969.
119. Magnusson C, Wedrén S, Rosenberg LU. Cigarette smoking and breast cancer risk: a population-based study in Sweden. Br J Cancer 2007; 97: 1287-1290.
120. Manjer J, Johansson R, Lenner P. Smoking is associated with postmenopausal breast cancer in women with high levels of estrogens. Int J Cancer 2004; 112: 324-328.
121. Martin-Moreno JM, Boyle P, Gorgojo L et al. Alcoholic beverage consumption and risk of breast cancer in Spain. Cancer Causes Control 1993; 4: 345-353.
122. McCredie MR, Dite GS, Giles GG, Hopper JL. Breast cancer in Australian women under the age of 40. Cancer Causes Control 1998; 9: 189-198.
123. McTiernan A, Thomas DB, Johnson LK, Roseman D. Risk factors for estrogen receptor-rich and estrogen receptor-poor breast cancers. J Natl Cancer Inst 1986; 77: 849-854.
124. Meara J, McPherson K, Roberts M et al. Alcohol, cigarette smoking and breast cancer. Br J Cancer 1989; 60: 70-73.
125. Millikan R, Pittman G, Tse CK et al. Glutathione S-transferases M1, T1, and P1 and breast cancer. Cancer Epidemiol Biomarkers Prev 2000; 9: 567-573.
126. Morabia A, Bernstein M, Héritier S, Khatchatrian N. Relation of breast cancer with passive and active exposure to tobacco smoke. Am J Epidemiol 1996; 143: 918-928.
127. Morabia A, Bernstein M, Ruiz J et al. Relation of smoking to breast cancer by estrogen receptor status. Int J Cancer 1998; 75: 339-342.
128. Morabia A, Bernstein MS, Bouchardy I et al. Breast cancer and active and passive smoking: the role of the N-acetyltransferase 2 genotype. Am J Epidemiol 2000; 152: 226-232.
129. Nishino Y, Minami Y, Kawai M et al. Cigarette smoking and breast cancer risk in relation to joint estrogen and progesterone receptor status: a case-control study in Japan. Springerplus 2014; 3: 65.
130. O'Connell DL, Hulka BS, Chambless LE et al. Cigarette smoking, alcohol consumption, and breast cancer risk. J Natl Cancer Inst 1987; 78: 229-234.
131. Ozmen V, Ozcinar B, Karanlik H et al. Breast cancer risk factors in Turkish women--a University Hospital based nested case control study. World J Surg Oncol 2009; 7: 37.
132. Palmer JR, Rosenberg L, Clarke EA et al. Breast cancer and cigarette smoking: a hypothesis. Am J Epidemiol 1991; 134: 1-13.
133. Pawlega J. Breast cancer and smoking, vodka drinking and dietary habits. A case-control study. Acta Oncol 1992; 31: 387-392.
134. Pięta B, Chmaj-Wierzchowska K, Opala T. Life style and risk of development of breast and ovarian cancer. Ann Agric Environ Med 2012; 19: 379-384.
135. Pimhanam C, Sangrajrang S, Ekpanyaskul C. Tobacco smoke exposure and breast cancer risk in Thai urban females. Asian Pac J Cancer Prev 2014; 15: 7407-7411.
136. Plagens-Rotman K, Piskorz-Szymendera M, Chmaj-Wierzychowska K, Pieta B. Breast cancer - Analysis of the selected risk factors. Eur J Gynaecol Oncol 2017; 38: 425-430.
137. Pongsavee M, Wisuwan K. ERCC5 rs751402 polymorphism is the risk factor for sporadic breast cancer in Thailand. Int J Mol Epidemiol Genet 2018; 9: 27-33.

138. Prescott J, Ma H, Bernstein L, Ursin G. Cigarette smoking is not associated with breast cancer risk in young women. Cancer Epidemiol Biomarkers Prev 2007; 16: 620-622.
139. Rabstein S, Brüning T, Harth V et al.; GENICA Network. N-acetyltransferase 2, exposure to aromatic and heterocyclic amines, and receptor-defined breast cancer. Eur J Cancer Prev 2010; 19: 100-109.
140. Rajaei M, Omidvari S, Saadat M. Influence of smoking habit on age at diagnosis of breast cancer. Serbian Journal of Experimental and Clinical Research 2015; 16: 213-216.
141. Ranstam J, Olsson H. Alcohol, cigarette smoking, and the risk of breast cancer. Cancer Detect Prev 1995; 19: 487-493.
142. Rautalahti M, Albanes D, Virtamo J et al. Lifetime menstrual activity--indicator of breast cancer risk. Eur J Epidemiol 1993; 9: 17-25.
143. Richardson S, de Vincenzi I, Pujol H, Gerber M. Alcohol consumption in a case-control study of breast cancer in southern France. Int J Cancer 1989; 44: 84-89.
144. Roddam AW, Pirie K, Pike MC et al. Active and passive smoking and the risk of breast cancer in women aged 36-45 years: a population based case-control study in the UK. Br J Cancer 2007; 97: 434-439.
145. Rohan TE, Baron JA. Cigarette smoking and breast cancer. Am J Epidemiol 1989; 129: 36-42.
146. Rollison DE, Brownson RC, Hathcock HL, Newschaffer CJ. Case-control study of tobacco smoke exposure and breast cancer risk in Delaware. BMC Cancer 2008; 8: 157.
147. Ruszczyk M, Zirpoli G, Kumar S et al. Breast cancer risk factor associations differ for pure versus invasive carcinoma with an in situ component in case-control and case-case analyses. Cancer Causes Control 2016; 27: 183-198.
148. Shore RE, Zeleniuch-Jacquotte A, Currie D et al. Polymorphisms in XPC and ERCC2 genes, smoking and breast cancer risk. Int J Cancer 2008; 122: 2101-2105.
149. Slattery ML, Curtin K, Giuliano AR et al. Active and passive smoking, IL6, ESR1, and breast cancer risk. Breast Cancer Res Treat 2008; 109: 101-111.
150. Smith SJ, Deacon JM, Chilvers CE. Alcohol, smoking, passive smoking and caffeine in relation to breast cancer risk in young women. UK National Case-Control Study Group. Br J Cancer 1994; 70: 112-119.
151. Stanford JL, Szklo M, Boring CC et al. A case-control study of breast cancer stratified by estrogen receptor status. Am J Epidemiol 1987; 125: 184-194.
152. Trivers KF, Lund MJ, Porter PL et al. The epidemiology of triple-negative breast cancer, including race. Cancer Causes Control 2009; 20: 1071-1082.
153. Tung HT, Tsukuma H, Tanaka H et al. Risk factors for breast cancer in Japan, with special attention to anthropometric measurements and reproductive history. Jpn J Clin Oncol. 1999; 29: 137-146.
154. Ueji M, Ueno E, Hyiaman DO et al. Risk Factors for Breast Cancer among Japanese Women: A Case-Control Study in Ibaraki, Japan. Breast Cancer 1998; 5: 351-358.
155. van der Hel OL, Peeters PH, Hein DW et al. NAT2 slow acetylation and GSTM1 null genotypes may increase postmenopausal breast cancer risk in long-term smoking women. Pharmacogenetics 2003; 13: 399-407.
156. van der Hel OL, Bueno-de-Mesquita HB, van Gils CH et al. Cumulative genetic defects in carcinogen metabolism may increase breast cancer risk (The Netherlands). Cancer Causes Control 2005; 16: 675-681.
157. Viladiu P, Izquierdo A, de Sanjosé S, Bosch FX. A breast cancer case-control study in Girona, Spain. Endocrine, familial and lifestyle factors. Eur J Cancer Prev 1996; 5: 329-335.
158. Wakai K, Ohno Y, Watanabe S et al. Risk Factors for Breast Cancer among Japanese Women in Tokyo : A Case-Control Study. J Epidemiol 1994; 4: 65-71.
159. Wang Q, Li H, Tao P et al. Soy isoflavones, CYP1A1, CYP1B1, and COMT polymorphisms, and breast cancer: a case-control study in southwestern China. DNA Cell Biol 2011; 30: 585-595.
160. White E, Malone KE, Weiss NS, Daling JR. Breast cancer among young U.S. women in relation to oral contraceptive use. J Natl Cancer Inst 1994; 86: 505-514.
161. Yang PS, Yang TL, Liu CL et al. A case-control study of breast cancer in Taiwan--a low-incidence area. Br J Cancer 1997; 75: 752-756.

162. Yoo KY, Tajima K, Miura S et al. Breast cancer risk factors according to combined estrogen and progesterone receptor status: a case-control analysis. Am J Epidemiol 1997; 146: 307-314.
163. Young E, Leatherdale S, Sloan M et al. Age of smoking initiation and risk of breast cancer in a sample of Ontario women. Tob Induc Dis 2009; 5: 4.
164. Yuan JM, Yu MC, Ross RK et al. Risk factors for breast cancer in Chinese women in Shanghai. Cancer Res 1988; 48: 1949-1953.
165. Zheng W, Deitz AC, Campbell DR et al. N-acetyltransferase 1 genetic polymorphism, cigarette smoking, well-done meat intake, and breast cancer risk. Cancer Epidemiol Biomarkers Prev 1999; 8: 233-239.
166. Zheng T, Holford TR, Zahm SH et al. Cigarette smoking, glutathione-s-transferase M1 and t1 genetic polymorphisms, and breast cancer risk (United States). Cancer Causes Control 2002; 13: 637-645.
167. Zheng H, Song F, Zhang L et al. Genetic variants at the miR-124 binding site on the cytoskeleton-organizing IQGAP1 gene confer differential predisposition to breast cancer. Int J Oncol 2011; 38: 1153-1161.
168. Akiba S, Hirayama T. Cigarette smoking and cancer mortality risk in Japanese men and women--results from reanalysis of the six-prefecture cohort study data. Environ Health Perspect 1990; 87: 19-26.
169. Al-Delaimy WK, Cho E, Chen WY et al. A prospective study of smoking and risk of breast cancer in young adult women. Cancer Epidemiol Biomarkers Prev 2004; 13: 398-404.
170. Arthur R, Wassertheil-Smoller S, Manson JE et al. The Combined Association of Modifiable Risk Factors with Breast Cancer Risk in the Women's Health Initiative. Cancer Prev Res (Phila) 2018; 11: 317-326.
171. Arthur RS, Wang T, Xue X et al. Genetic factors, adherence to healthy lifestyle behavior, and risk of invasive breast cancer among women in the UK Biobank. J Natl Cancer Inst 2020; 112(9):893-901.
172. Blakely T, Barendregt JJ, Foster RH et al. The association of active smoking with multiple cancers: national census-cancer registry cohorts with quantitative bias analysis. Cancer Causes Control 2013; 24: 1243-1255.
173. Calle EE, Miracle-McMahill HL, Thun MJ, Heath CW, Jr. Cigarette smoking and risk of fatal breast cancer. Am J Epidemiol 1994; 139: 1001-1007.
174. Carter BD, Abnet CC, Feskanich D et al. Smoking and mortality--beyond established causes. N Engl J Med 2015; 372: 631-640.
175. Coleman NC, Burnett RT, Higbee JD et al. Cancer mortality risk, fine particulate air pollution, and smoking in a large, representative cohort of US adults. Cancer Causes Control 2020; 31(8):767-776.
176. Engeland A, Andersen A, Haldorsen T, Tretli S. Smoking habits and risk of cancers other than lung cancer: 28 years' follow-up of 26,000 Norwegian men and women. Cancer Causes Control 1996; 7: 497-506.
177. Gaudet MM, Gapstur SM, Sun J et al. Active smoking and breast cancer risk: original cohort data and meta-analysis. J Natl Cancer Inst 2013; 105: 515-525.
178. Goodman MT, Cologne JB, Moriwaki H et al. Risk factors for primary breast cancer in Japan: 8-year follow-up of atomic bomb survivors. Prev Med 1997; 26: 144-153.
179. Gram IT, Braaten T, Terry PD et al. Breast cancer risk among women who start smoking as teenagers. Cancer Epidemiol Biomarkers Prev 2005; 14: 61-66.
180. Gram IT, Park SY, Kolonel LN et al. Smoking and Risk of Breast Cancer in a Racially/Ethnically Diverse Population of Mainly Women Who Do Not Drink Alcohol: The MEC Study. Am J Epidemiol 2015; 182: 917-925.
181. Gram IT, Park SY, Maskarinec G et al. Smoking and breast cancer risk by race/ethnicity and oestrogen and progesterone receptor status: the Multiethnic Cohort (MEC) study. Int J Epidemiol 2019; 48: 501-511.
182. Hanaoka T, Yamamoto S, Sobue T et al; Japan Public Health Center-Based Prospective Study on Cancer and Cardiovascular Disease Study Group. Active and passive smoking and breast cancer risk in middle-aged Japanese women. Int J Cancer 2005; 114: 317-322.
183. Hiatt RA, Fireman BH. Smoking, menopause, and breast cancer. J Natl Cancer Inst 1986; 76: 833-838.

184. Jacob L, Freyn M, Kalder M et al. Impact of tobacco smoking on the risk of developing 25 different cancers in the UK: a retrospective study of 422,010 patients followed for up to 30 years. Oncotarget 2018; 9: 17420-17429.
185. Jee SH, Samet JM, Ohrr H et al. Smoking and cancer risk in Korean men and women. Cancer Causes Control 2004; 15: 341-348.
186. Jones ME, Schoemaker MJ, Wright LB et al. Smoking and risk of breast cancer in the Generations Study cohort. Breast Cancer Res 2017; 19: 118.
187. Ko KP, Kim SJ, Huzarski T et al.; Hereditary Breast Cancer Clinical Study Group. The association between smoking and cancer incidence in BRCA1 and BRCA2 mutation carriers. Int J Cancer 2018; 142: 2263-2272.
188. Land SR, Liu Q, Wickerham DL et al. Cigarette smoking, physical activity, and alcohol consumption as predictors of cancer incidence among women at high risk of breast cancer in the NSABP P-1 trial. Cancer Epidemiol Biomarkers Prev 2014; 23: 823-832.
189. Lin Y, Kikuchi S, Tamakoshi K et al.; Japan Collaborative Cohort Study Group for Evaluation of Cancer Risk. Active smoking, passive smoking, and breast cancer risk: findings from the Japan Collaborative Cohort Study for Evaluation of Cancer Risk. J Epidemiol 2008; 18: 77-83.
190. London SJ, Colditz GA, Stampfer MJ et al. Prospective study of smoking and the risk of breast cancer. J Natl Cancer Inst 1989; 81: 1625-1631.
191. Luo J, Margolis KL, Wactawski-Wende J et al. Association of active and passive smoking with risk of breast cancer among postmenopausal women: a prospective cohort study. BMJ 2011; 342: d1016.
192. Manjer J, Berglund G, Bondesson L et al. Breast cancer incidence in relation to smoking cessation. Breast Cancer Res Treat 2000; 61: 121-129.
193. Manjer J, Malina J, Berglund G et al. Smoking associated with hormone receptor negative breast cancer. Int J Cancer 2001; 91: 580-584.
194. Meyer J, Rohrmann S, Bopp M et al.; Swiss National Cohort Study Group. Impact of Smoking and Excess Body Weight on Overall and Site-Specific Cancer Mortality Risk. Cancer Epidemiol Biomarkers Prev 2015; 24: 1516-1522.
195. Nordlund LA, Carstensen JM, Pershagen G. Cancer incidence in female smokers: a 26-year follow-up. Int J Cancer 1997; 73: 625-628.
196. Nyante SJ, Gierach GL, Dallal CM et al. Cigarette smoking and postmenopausal breast cancer risk in a prospective cohort. Br J Cancer 2014; 110: 2339-2347.
197. Olson JE, Vachon CM, Vierkant RA et al. Prepregnancy exposure to cigarette smoking and subsequent risk of postmenopausal breast cancer. Mayo Clin Proc 2005; 80: 1423-1428.
198. Ozasa K, Japan Collaborative Cohort Study for Evaluation of Cancer. Smoking and mortality in the Japan Collaborative Cohort Study for Evaluation of Cancer (JACC). Asian Pac J Cancer Prev 2007; 8 Suppl: 89-96.
199. Park B, Lim SE, Ahn H et al. Heterogenous Effect of Risk Factors on Breast Cancer across the Breast Density Categories in a Korean Screening Population. Cancers (Basel) 2020; 12.
200. Pirie K, Peto R, Reeves GK et al.; Million Women Study Collaborators. The 21st century hazards of smoking and benefits of stopping: a prospective study of one million women in the UK. Lancet 2013; 381: 133-141.
201. Reynolds P, Hurley S, Goldberg DE et al. Active smoking, household passive smoking, and breast cancer: evidence from the California Teachers Study. J Natl Cancer Inst 2004; 96: 29-37.
202. Rosenberg L, Boggs DA, Bethea TN et al. A prospective study of smoking and breast cancer risk among African-American women. Cancer Causes Control 2013; 24: 2207-2215.
203. Schatzkin A, Carter CL, Green SB et al. Is alcohol consumption related to breast cancer? Results from the Framingham Heart Study. J Natl Cancer Inst 1989; 81: 31-35.
204. Swift M, Lukin JL. Breast cancer incidence and the effect of cigarette smoking in heterozygous carriers of mutations in the ataxia-telangiectasia gene. Cancer Epidemiol Biomarkers Prev 2008; 17: 3188-3192.
205. Taghizadeh N, Vonk JM, Boezen HM. Lifetime Smoking History and Cause-Specific Mortality in a Cohort Study with 43 Years of Follow-Up. PLoS One 2016; 11: e0153310.
206. Tverdal A, Thelle D, Stensvold I et al. Mortality in relation to smoking history: 13 years' follow-up of 68,000 Norwegian men and women 35-49 years. J Clin Epidemiol 1993; 46: 475-487.

207. van den Brandt PA. A possible dual effect of cigarette smoking on the risk of postmenopausal breast cancer. Eur J Epidemiol 2017; 32: 683-690.
208. Viner B, Barberio AM, Haig TR et al. The individual and combined effects of alcohol consumption and cigarette smoking on site-specific cancer risk in a prospective cohort of 26,607 adults: results from Alberta's Tomorrow Project. Cancer Causes Control 2019; 30: 1313-1326.
209. Wada K, Kawachi T, Hori A et al. Husband's smoking status and breast cancer risk in Japan: From the Takayama study. Cancer Sci 2015; 106: 455-460.
210. White AJ, D'Aloisio AA, Nichols HB et al. Breast cancer and exposure to tobacco smoke during potential windows of susceptibility. Cancer Causes Control 2017; 28: 667-675.
211. Zheng W, McLerran DF, Rolland BA et al. Burden of total and cause-specific mortality related to tobacco smoking among adults aged >/= 45 years in Asia: a pooled analysis of 21 cohorts. PLoS Med 2014; 11: e1001631.
